# Filling the Gaps in the Cyanobacterial Tree of Life—Metagenome Analysis of *Stigonema ocellatum* DSM 106950, *Chlorogloea purpurea* SAG 13.99 and *Gomphosphaeria aponina* DSM 107014

**DOI:** 10.3390/genes12030389

**Published:** 2021-03-09

**Authors:** Pia Marter, Sixing Huang, Henner Brinkmann, Silke Pradella, Michael Jarek, Manfred Rohde, Boyke Bunk, Jörn Petersen

**Affiliations:** 1 Leibniz-Institut DSMZ—Deutsche Sammlung von Mikroorganismen und Zellkulturen, 38124 Braunschweig, Germany; pia.marter@dsmz.de (P.M.); sixing.huang@dsmz.de (S.H.); henner.brinkmann@dsmz.de (H.B.); silke.pradella@dsmz.de (S.P.); boyke.bunk@dsmz.de (B.B.); 2 Helmholtz-Zentrum für Infektionsforschung, 38124 Braunschweig, Germany; michael.jarek@helmholtz-hzi.de (M.J.); manfred.rohde@helmholtz-hzi.de (M.R.)

**Keywords:** metagenome binning, phylogeny, text mining, classification, cyanosphere, non-axenic, microbial dark matter, culture collection

## Abstract

Cyanobacteria represent one of the most important and diverse lineages of prokaryotes with an unparalleled morphological diversity ranging from unicellular cocci and characteristic colony-formers to multicellular filamentous strains with different cell types. Sequencing of more than 1200 available reference genomes was mainly driven by their ecological relevance (*Prochlorococcus*, *Synechococcus*), toxicity (*Microcystis*) and the availability of axenic strains. In the current study three slowly growing non-axenic cyanobacteria with a distant phylogenetic positioning were selected for metagenome sequencing in order to (i) investigate their genomes and to (ii) uncover the diversity of associated heterotrophs. High-throughput Illumina sequencing, metagenomic assembly and binning allowed us to establish nearly complete high-quality draft genomes of all three cyanobacteria and to determine their phylogenetic position. The cyanosphere of the limnic isolates comprises up to 40 heterotrophic bacteria that likely coexisted for several decades, and it is dominated by *Alphaproteobacteria* and *Bacteriodetes*. The diagnostic marker protein RpoB ensured in combination with our novel taxonomic assessment via BLASTN-dependent text-mining a reliable classification of the metagenome assembled genomes (MAGs). The detection of one new family and more than a dozen genera of uncultivated heterotrophic bacteria illustrates that non-axenic cyanobacteria are treasure troves of hidden microbial diversity.

## 1. Introduction

The pivotal capacity of oxygenic photosynthesis originated in the common ancestor of all cyanobacteria more than 3.5 billion years ago [1] and it was subsequently acquired via primary, secondary and tertiary endosymbiosis by various lineages of eukaryotic algae and land plants [2,3]. Cyanobacteria were the first lineage of organisms that irreversibly remodeled the atmosphere of our planet by the release of toxic oxygen [4], but they are still among the most important primary producers in marine and limnic ecosystems [5]. In natural habitats, they live in close association with heterotrophic bacteria and their axenization is a challenging task [6], which depends on the tight physical cell–cell contact and mutualistic interactions [7]. The ecological interface for phytoplankton-bacteria relationships has been designated as “phycosphere” in analogy to the plant rhizosphere [8], and the term “cyanosphere” has been introduced to describe cyanobacterial microbiomes [9]. Cyanobacteria represent one of the most important and diverse bacterial phyla, but exhibit the probably worst taxonomy among all prokaryotic lineages. The reason is a four-decade-long jurisdictional conflict between the International Code of Nomenclature for algae, fungi, and plants (ICN; “Botanical Code”) and the International Code of Nomenclature of Prokaryotes (ICNP; “Prokaryotic Code”) that ended up in a nomenclatural deadlock [10].

The current understanding of microbial biodiversity is largely driven by the technical progress in nucleic acid sequencing and bioinformatics [11,12]. The genetic potential of uncultivated bacteria was already explored by biologists and chemists in the early genome era more than twenty years ago, when metagenomic soil DNA was cloned into bacterial artificial chromosomes and the resulting *Escherichia coli* transformants were screened for promising natural products [13]. Analyses of 16S rRNA gene sequences from uncultivated microbiota provided first insights into the hidden diversity of *Bacteria* and *Archaea* in the environment [14,15]. The development of innovative and comparably cheap next-generation sequencing technologies (Roche 454, Illumina; [16]) paved the way for environmental metagenome analyses. Initial high throughput sequencing studies of environmental samples revealed the genetic repertoire of marine and terrestrial communities from seawater, soil and biofilms [17,18,19]. However, the de novo genome assembly based on short-read sequences is limited even for isolated bacteria by duplicated stretches of DNA. Multiple copies of transposable elements or rRNA operons [20] thus account for a typical number of 30 to 200 contigs per draft genome. Large microbial metagenomes may comprise hundreds of thousands of contigs whose relationships and taxonomic affiliation remained largely unclear for more than a decade. This problem has meanwhile been solved by a smart bioinformatic strategy, the so-called metagenomic binning approach [21,22,23], which uses diagnostic genetic imprints of each species together with the relative abundance of all contigs in the metagenome to reconstruct individual genomes with a very good accuracy [24]. The quality of the resulting metagenome-assembled genomes (MAGs) can be estimated by three diagnostic criteria, (i) the assembly characteristics including the presence of the 16S rRNA gene, (ii) the completeness of the MAG and (iii) its contamination level [25,26]. Accordingly, meta- and single cell genomics boosted the knowledge about microbial diversity on our planet and recently resulted in a controversial discussion about the development of consistent rules for the nomenclature of uncultivated taxa [27,28].

The history, current state and future perspectives of (meta-)genomic approaches in cyanobacterial research are summarized in a review article by Alvarenga and colleagues [29]. The associated heterotrophs of non-axenic cyanobacteria were traditionally regarded as nasty contaminants and thus not further considered, which is exemplified for genome sequencing of the mat-forming diazotroph *Geitlerinema* sp. PCC 9228 (synonym *Oscillatoria limnetica* “Solar Lake”) [30]. In contrast, a seminal metagenome study about annual freshwater blooms of the diazotroph *Aphanizomenon flos-aquae*, which was based on Illumina short-read and PacBio long-read sequencing, resulted in complete chromosome assemblies of three dominant associated bacteria representing an α- and a betaproteobacterium as well as a *Bacteroidetes* strain [31]. The neglected relevance of these bioresources was recently uncovered by the group of Denis Baurain, who investigated more than a dozen non-axenic (sub)polar cyanobacteria of the Belgian BCCM/ULC collection with a novel metagenomic pipeline [32]. All cyanobacterial MAGs showed more than 90% of completeness and a contamination level below 2%, which is comparable to those of axenic cyanobacteria [33,34]. Phylogenomic analyses allowed them to determine the positioning of the (sub)polar strains in the cyanobacterial tree of life. Moreover, they revealed very close relationships of associated bacteria from geographically distant sampling sites [32], which might reflect specific phototroph-heterotroph interactions in the cyanosphere.

The spectrum of genome-sequenced cyanobacteria essentially reflects (i) the cultivability of species and (ii) the axenicity of available strains. In the light of their great diversity [35], many cyanobacterial lineages have thus not been investigated so far [36]. To fill some of the gaps in the cyanobacterial tree of life, we compared a set of more than 800 cyanobacteria, which are deposited at the German Collection of Microorganisms and Cell Cultures (DSMZ) or the Culture Collection of Algae in Göttingen (SAG), and identified three non-axenic strains belonging to different cyanobacterial families that have so far not been molecularly analyzed beyond the 16S rRNA gene level. The first goal of our current study was the establishment and analysis of the MAGs of the respective strains *Stigonema ocellatum* DSM 106950 (*Stigonemataceae*), *Gomphosphaeria aponina* DSM 107014 (*Gomphosphaeriaceae*) and *Chlorogloea purpurea* SAG 13.99 (*Entophysalidaceae*). The second goal was a metagenome-based characterization of their low complexity communities in terms of composition and taxonomy.

## 2. Material and Methods

### 2.1. Strains and Cultivation Conditions

*S. ocellatum* DSM 106950 (=SAG 48.90) and *G. aponina* DSM 107014 (=SAG 52.96) are available at the DSMZ (German Collection of Microorganisms and Cell Cultures, Braunschweig, Germany; Table 1), and *C. purpurea* SAG 13.99 was kindly provided by the Sammlung von Algenkulturen der Universität Göttingen (Culture Collection of Algae at Göttingen University, SAG, Germany). *Stigonema* was cultivated in 20 mL Z45/4 medium (DSMZ medium 1727), *Chlorogloea* in ES medium (DSMZ medium 1693) and *Gomphosphaeria* in Z medium (DSMZ medium 1726) in 25 cm^2^ tissue culture flasks (Techno Plastic Products AG [TPP], Trasadingen, Switzerland). The strains grew at 17 °C under low light conditions (3–4 µmol s^−1^ m^−2^) at a day/night cycle of 16 h/8 h. All three cyanobacteria were successfully cryopreserved in 9% DMSO (Sigma-Aldrich, Darmstadt, Germany) as previously described [33].

### 2.2. Light and Electron Microscopy

For light microscopy cyanobacteria were fixed in 1% glutaraldehyde (Sigma-Aldrich, Darmstadt, Germany) at 4 °C overnight. Subsequently, cells were washed three times with PBS and were analyzed with the inverse Nikon microscope Eclipse Ti-E (Nikon, Tokyo, Japan) as previously described [33]. For scanning electron microscopy (SAM) with the Zeiss Merlin field emission scanning electron microscope (Carl Zeiss, Oberkochen, Germany) cyanobacteria were fixed in the cultivation medium with glutaraldehyde (final concentration 2%). The final fixation was achieved after 30 min by adding formaldehyde (Riedel-de Haën, Seelze, Germany) to a final concentration of 5%. SAM was performed as previously described [33].

### 2.3. Metagenomics

#### 2.3.1. Metagenome Sequencing

DNA was extracted with the DNeasy^®^ Blood and Tissue Kit (Qiagen, Hilden, Germany) with a pretreatment recommended for Gram-positive bacteria. The protocol was modified by performing the initial enzymatic lysis step of the non-axenic cyanobacteria with lysozyme in the provided lysis buffer over-night at 37 °C. The usage of sterile 600 µm glass beads and shaking on the Eppendorf^®^ thermomixer compact (Hamburg, Germany) with 600 rpm resulted in comparably harsh extraction conditions that should ensure the isolation of DNA from all associated microbes. Total amounts of 0.5 µg, 10.3 µg and 6.8 µg DNA were isolated from *S. ocellatum* DSM 106950, *C. purpurea* SAG 13.99 and *G. aponina* DSM 107014, respectively. Illumina libraries were prepared using the NEBNext Ultra II FS DNA Library Prep Kit (New England Biolabs, Frankfurt, Germany) according to the instructions of the manufacturer. Sequencing of the libraries was performed on the Illumina NovaSeq 6000 system using the v3 chemistry (600 cycles) following standard protocol. Quality control and adapter clipping of the sequences was done using fastq-mcf tool of ea-utils v1.04.803 [37].

#### 2.3.2. Metagenome Assembly and Binning

Sequence data were first trimmed by the tool Sickle (Joshi and Fass 2011: Sickle: A sliding-window, adaptive, quality-based trimming tool for FastQ files [Software version 1.33 available at https://github.com/najoshi/sickle] (last accessed on 21 December 2020)) with default parameters. Sequence data were filtered against non-authentic primer and adapter sequences originating from the Illumina library preparation (“AATGATACGGCGACCACCGAGATCT”, “GTATGCCGTCTTCTGC”, “AAGAGCGTCGTGTAGGGAAAGA” and “GATCGGAAGAGCACACGTCTGAACTCCAGTCAC”) by a custom Python script (https://github.com/dgg32/maxbin2_checkm_slurm_illumina (last accessed on 21 December 2020)). This step is crucial to avoid misleading results from subsequent text mining analyses, designated as the “carp artifact” (see Supplemental Text File S1A). The filtered and trimmed sequences were uploaded to NCBI’s Sequence Read Archive (Accession numbers: SRR12487250, SRR12487251, SRR12487252). The reads were assembled with MEGAHIT v1.2.7 [38] with default parameters. The assembled sequences were subsequently binned with MaxBin 2.0 v2.2.6 [21], MetaBAT v2.12.1 [23] and Concoct v1.1.0 [22] (with Bowtie version 2.3.5 [39]) with default parameters. All three sets of binned metagenomes were subsequently analyzed with DAS Tool v1.1.2 [40]. The resulting bins were investigated with CheckM v1.0.13 with the lineage_wf option [26]. Afterwards, the cyanobacterial bins were manually curated based on first, the results obtained from the comparison against the NCBI nt database (accessed: 11 December 2019) per BLASTN v2.4.0 and second, the 16S rDNA sequences in the datasets. Afterwards, the bins were checked again with CheckM. Coverage values were calculated per bbmap (last modified February 11, 2019). The genomes were annotated by DFAST v1.2.6 [41], InterProScan v5.48-83.0 [42] and SignalP v5.0 [43].

### 2.4. Taxonomic Assessment via BLASTN-Dependent Text Mining

Text mining for the rapid taxonomic assessment of metagenomic bins was performed via BLASTN searches of all assembled DNA contigs against the NCBI nt database (fetched at 11 December 2019). The Subject title “stitle” sections of the 20 best hits for each contig were considered if their e-values were lower than 1 × 10^−10^ and identities were higher than 90%. Finally, the most common 20 words of all contigs per bin were counted ignoring “complete”, “genome”, “DNA”, “sequence”, “sp.”, “strain” and “assembly” since they do not contain relevant taxonomic information. The results were manually inspected.

### 2.5. Phylogenetic Analyses

CheckM [26], which already allows a very rough taxonomic classification of the analyzed bins with the identified “marker lineages”, was used to retrieve the set of 43 universal proteins from 213 cyanobacterial genomes and to generate an alignment of the concatenated protein sequences. Manual refinement of the alignment with the MUST package [44], application of G-blocks [45] and the calculation of maximum likelihood (ML) trees with RAxML v8.2.10 [46] was conducted as previously described [33].

### 2.6. Manual Curation of Metagenome Assembled Genomes (MAGs)

Cyanobacterial MAGs derived from MaxBin were manually curated prior to GenBank submission by (i) the removal or addition of contigs comprising wrongly binned rRNA and tRNA genes, (ii) the addition of cyanobacterial contigs that were exclusively detected with Concoct in combination with our text mining approach and (iii) in the case of *C. chlorogloea* SAG 13.99 the addition of a secondary bin comprising putative plasmid DNA. In detail, the MAGs have been curated as follows: (1) *S. ocellatum* DSM 106950 (GenBank Accession: JADQBA000000000; 509 contigs). Cyanobacterial genome: Bin01 (516 contigs); removal of two 16S-rRNA, two 23S-rRNA, one 5S-rRNA and three tRNA contigs; addition of one 23S-rRNA contig from bin40. (2) *C. purpurea* SAG 13.99 (GenBank Accession: JADQBB000000000; 228 contigs). Cyanobacterial genome: Bin02 [marker genes] and bin01 [accessory genes] (229 contigs); removal of one 23S-rRNA and one tRNA contig; addition of one 16S-rRNA plus 23S-rRNA contig from bin34. (3) *G. aponina* DSM 107014 (GenBank Accession: JADQBC000000000; 749 contigs). Cyanobacterial genome: Bin03 (748 contigs); removal of one tRNA contig; addition of one 16S-rRNA contig and one 23S-rRNA contig from bin20.

## 3. Results

### 3.1. Light and Scanning Electron Microscopy

**Figure 1:**
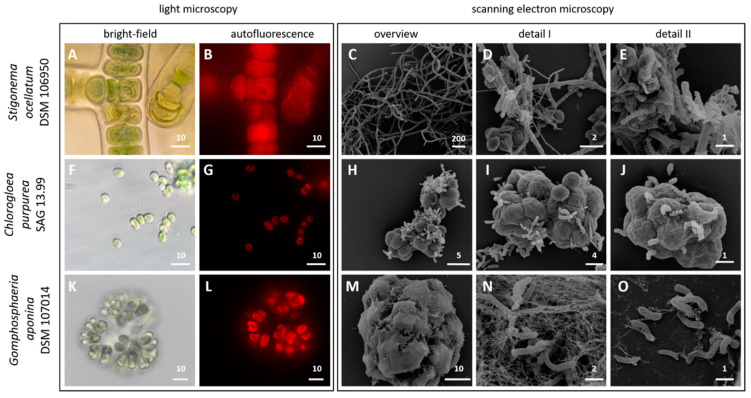
Light and scanning electron microscopy of three non-axenic cyanobacteria, i.e., *Stigonema ocellatum* DSM 106950 (**A**–**E**), *Chlorogloea purpurea* SAG 13.99 (**F**–**J**) and *Gomphosphaeria aponina* DSM 107014 (**K**–**O**). Scale bar for light microscopy: 10 µm (**A**,**B**,**F**,**G**,**K**,**L**). Scale bars for SEM overview: 200 µm (**C**), 5 µm (**H**), 10 µm (**M**); detail I: 2 µm (**D**), 4 µm (I), 2 µm (N) and detail II: 1 µm (**E**,**J**,**O**).

Cell division of unicellular or colony-forming cyanobacteria and the branching pattern of filamentous strains once served as diagnostic traits for their taxonomic classification in five sections [6]. The characteristic morphology of the three cyanobacteria investigated in the current study is shown in Figure 1. The light microscopic image of *S. ocellatum* DSM 106950 (=SAG 48.90) illustrates that it is a multicellular representative of the T-branching type (Table 1). The specific heart-shaped cells of *G. aponina* DSM 107014 (=SAG 52.96) are connected via central mucilaginous stalks and form characteristic colonies (Figure 1). Light microscopy confirmed that *C. purpurea* SAG 13.99 is a unicellular cyanobacterium with spherical and sometimes attached cells.

The scanning electron micrographs of the three non-axenic cyanobacteria illustrate a great morphological diversity of associated microbes (Figure 1, Figure S1). The cyanosphere of *S. ocellatum* DSM 106950 contains various coccoid, rod-shaped and helical bacteria (Figure 1C–E). The attachment of associated bacteria on the cyanobacterial surface is best shown for aggregated *C. purpurea* SAG 13.99 cells that are surrounded by a fibrous sheath (Figure 1H–J). Some bacteria form stalks that mediate the cell–cell contact with the cyanobacterial host and other heterotrophs. Another mode of interaction is shown for the *G. aponina* DSM 107014 colonies, where the heterotrophs are imbedded into the thick surrounding matrix (Figure 1M–O). Our investigation of three morphologically and phylogenetically very distinct cyanobacteria provided a first glimpse into the formation of micro-biofilms on phototrophic bacteria and their complex networks. The tight association of various bacteria illustrates the challenges of a successful axenization especially of colony-forming and filamentous cyanobacteria [6]. It is remarkable that these non-axenic cyanobacteria exhibit a very complex and probably genuine community composition even decades after their isolation from the natural habitat. A prime example is *S. ocellatum* that was isolated by the German phycologist Dieter Mollenhauer from a *Sphagnum*-bog fifty years ago (Table 1).

### 3.2. Metagenome Sequencing of Three Non-Axenic Freshwater Cyanobacteria

Sequencing of total DNA from the non-axenic cyanobacteria on the Illumina NovaSeq platform provided the basis for the establishment of a nearly complete genome of the respective phototroph and the most abundant associated heterotrophs. The final metagenomic libraries had insert sizes between 650 bp and 700 bp. Primer-dimer filtering was an important step to remove non-authentic sequences from the preparation of the Illumina libraries (see Materials and Methods). The binning results of MaxBin were used as a basis for our further analyses according to the main goal of our study, i.e., the establishment of cyanobacterial MAGs that are as complete as possible (see Section 3.3). The size of the three metagenomes ranges from 5.9 Gbp (*Gomphospheria*), 22.0 Gbp (*Chlorogloea*) to 55.0 Gbp (*Stigonema*). The established metagenomes of *G. aponina, C. purpurea* and *S. ocellatum* contain 27, 45 and 44 bins, respectively (Table S1). The mapping of the filtered Illumina data on the MAGs allowed the calculation of the average coverage of each MAG ranging from a marginal value below 2 to more than 600 genome equivalents. It is noteworthy that, e.g., the flavobacterial bin32 from *C. purpurea* obtained a completeness of 99.15% and a contamination level of only 1.30% despite of its low coverage (5×). The authentic cyanobacterial MAGs of all three strains were found among the three most abundant bins of each dataset. Their genome completeness of at least 98.28% and the low contamination level (≤2.35%), which is comparable to those of axenic cultures with up to 5% allegedly detected false positive “contaminations” [33], documents the applicability of our sequencing and binning approach. High throughput Illumina NovaSeq sequencing of the three non-axenic cyanobacteria met two of three criteria for high-quality draft MAGs [25], i.e., a completeness of more than 90% and a contamination below 5%. The third criterion that requires the presence of the 16S, 23S and 5S rRNA genes is generally difficult to meet by the short-read sequencing approach. Accordingly, in our initial cyanobacterial assemblies only the *Stigonema* bin01 contained the 16S-rRNA gene (Table S2), which reflects the typical binning outcome of ribosomal operons comprising in comparison with protein-encoding genes a deviant nucleotide composition due to selective constraints and multiple copies per genome [20]. However, we succeeded to identify the authentic 16S and 23S rRNA genes from each cyanobacterium in the respective metagenome and our manual curation of the bins resulted in three high-quality draft MAGs (Table 1).

### 3.3. Comparison of the Binning Programs MaxBin, Concoct and MetaBAT

**Figure 2:**
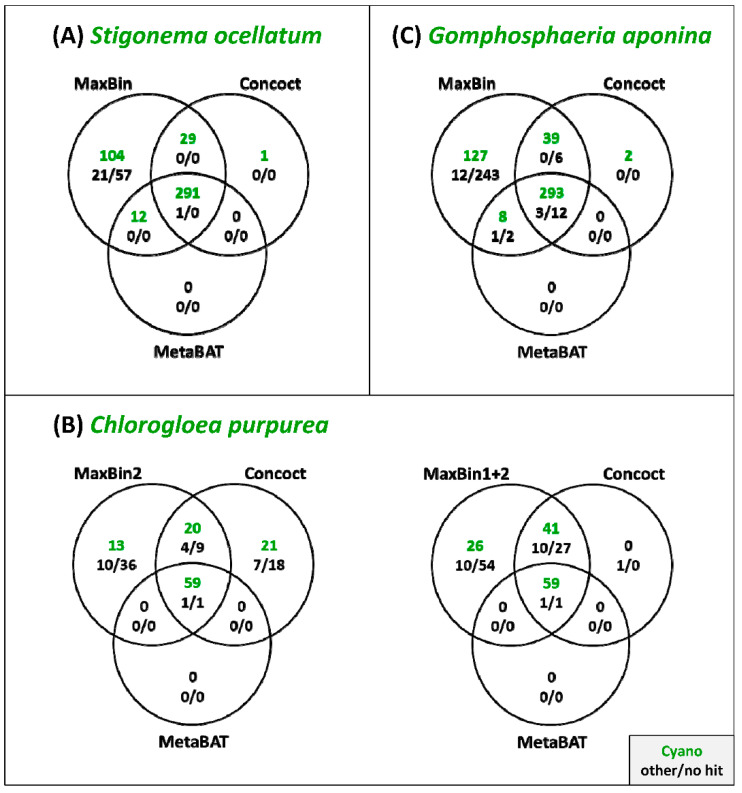
Comparison of cyanobacterial metagenome bins obtained with MaxBin, Concoct and MetaBAT. (**A**) *S. ocellatum* DSM 106950, (**B**) *C. purpurea* SAG 13.99, (**C**) *G. aponina* DSM 107014. The taxonomic affiliation of all contigs was determined with BLASTN searches, which allowed a differentiation between cyanobacterial (Cyano), non-cyanobacterial (other) and contigs without BLAST-results (no hit). Authentic cyanobacterial contigs are highlighted in green.

The main goal of the current study was the establishment of cyanobacterial MAGs to fill the gaps in the cyanobacterial tree of life. Dealing with non-axenic, but uni-cyanobacterial cultures is in comparison to environmental samples a great advantage, because it allows a reliable identification of authentic contigs based on BLAST analyses. We investigated our metagenome assemblies of *S. ocellatum*, *C. purpurea* and *G. aponina* with MaxBin, Concoct and MetaBAT [21,22,23], and evaluated the binning accuracy of the cyanobacterial MAGs (Table S1). According to the CheckM criteria, all three programs retrieved cyanobacterial bins of very good quality with a completeness of more than 93% and contamination levels below 3%. It is noteworthy that the complete cyanobacterial genome of *C. purpurea* presented by MaxBin is distributed on two separate bins. Bin02 likely represents the chromosome with essential marker genes and bin01 consists of plasmid sequences with accessory genes (see also Supplemental Text File S1B). The results from all three binning programs were further analyzed with DAS Tool [40]. This program dereplicates, aggregates and scores the contigs and should result in more accurate binnings. However, its cyanobacterial bin is for all three metagenomes an exact copy of the MetaBAT result, which is the least complete among the three methods (Table S1). The Venn diagrams in Figure 2 illustrate the unique and shared bins of the different binning approaches, and they clearly demonstrate the strength of MaxBin for the identification of genuine cyanobacterial contigs that are highlighted in green. Their authenticity was confirmed via BLASTN. The most conservative binning program MetaBAT could only detect between 47% and 69% of these cyanobacterial contigs, Concoct had a higher predictive ability of at least 71% and was finally outperformed by MaxBin with a forecasting power of more than 99%. MaxBin could specifically detect a total of 104, 26 and 127 cyanobacterial contigs of *S. ocellatum*, *C. purpurea* and *G. aponina*, respectively (Figure 2). The comparison of the three methods revealed a universal core set of contigs, but the “binning philosophy” of each tool led to a different pan contig set with a maximal recruitment strategy of MaxBin. For sake of completeness, one additional *Stigonema* and two *Gomphosphaeria* contigs, which could exclusively be recruited by Concoct, were manually added to the cyanobacterial MAGs obtained by MaxBin. Taken together, our analyses showed that the quality of automated metagenome binning can be substantially improved by external validation criteria.

### 3.4. Genome Properties of S. ocellatum, C. purpurea and G. aponina

The properties of the three manually curated high-quality MAGs are shown in Table 2. The filamentous *Stigonemataceae S. ocellatum* contains with 10.35 Mbp the largest genome of the investigated strains (JADQBA000000000), which is in comparison to the currently largest cyanobacterial genome of 12.29 Mbp in *Scytonema hofmannii* PCC 7110 (NZ_ANNX00000000.2) a remarkable size. The genomes of the unicellular or colonial strains *C. purpurea* (4.74 Mbp; JADQBB000000000) and *G. aponina* (5.34 Mbp; JADQBC000000000) are much smaller and comparable to those of the model organism *Synechocystis* sp. PCC 6803 with a genome size of 3.95 Mbp (GCA_000009725.1). The sparseness of assembled RNA genes in our MAGs reflects a characteristic problem of short-read sequences. The calculated gene versus genome ratios revealed an average proportion of 1173 bp per gene for *S. ocellatum*, 1070 bp for *C. purpurea* and 1006 bp for *G. aponina*, which might reflect an individual selection towards genome extension or streamlining. The total number of genes in *S. ocellatum* (8824), *C. purpurea* (4429) and *G. aponina* (5305) corresponds to their genome sizes (Table 2). *S. ocellatum* comprises about twice as many genes as *C. purpurea*, which reciprocally correlates with a proportion of genes with functional prediction of 32% and 44%, respectively. This conspicuous difference does in turn also document that about 6000 genes of *Stigonema*, representing more than two thirds of its genome, are yet not functionally characterized, which shows the great hidden genetic potential of this true branching cyanobacterium.

### 3.5. Discovery of Novel Bacterial Taxa in the Cyanosphere Based on the 16S-rRNA Gene

We used the 16S-rRNA gene for the taxonomic assessment of the bacterial diversity and the identification of novel taxa in the cyanosphere, because this marker still represents the gold standard for the classification of cultivated and uncultivated bacteria [47]. BLASTN searches of (nearly) complete 16S-rDNA sequences provided promising results for all three investigated metagenomes suggesting the presence of several new bacterial taxa that are living in close association with the respective cyanobacterium (Table S2). One example is the 1471 bp sequence from the *C. purpurea* bin44 that shares only a sequence similarity of 89.53% with the next relative *Burkholderia tropica* (KM97466.1). This 16S-rRNA gene is according to the 94.5% threshold criterion of Yarza et al. (2014) indicative of a bacterium representing a new genus within the family *Burkholderiaceae* (*Betaproteobacteria*). Another example is the 1497 bp 16S-rRNA sequence of the *G. aponina* bin08 that likely represents a new genus within the *Hymenobacteraceae* (*Bacteroidetes*) due to a similarity of 88.11% with its next relative *Adhaeribacter* sp. R-68225 (KY386541.1). However, the most conspicuous finding is the 16S-rDNA sequence of the *S. ocellatum* bin40 (1418 bp) that exhibits a similarity of only 82.02% with *Dehalogenimonas lykanthroporepellens* BL-DC-9 (CP002084.1), thus proposing the presence of a new family within the *Dehalococcoidia* (*Chloroflexi*; threshold 86.5% [47]). In total the 16S-rDNA analysis suggests the presence of at least 13 novel bacterial genera and one new family in the cyanosphere of the three investigated non-axenic strains (Table S2). The authenticity of the de novo-assembled 16S-rRNA genes should be experimentally cross-checked for follow-up studies, but BLASTN searches of bisected 16S-rDNA sequences, which contained either the 5′ or the 3′ half of the gene, provided no hint for the presence of sequence chimera.

### 3.6. Classification of Metagenomic Bins from Non-Axenic Cyanobacteria

The wealth of up to 45 bins from the three cyanobacterial metagenomes, which were established with MaxBin, raised the question about the taxonomic affiliation of the associated heterotrophs. The taxonomic assessment of the bins into different “marker lineages” based on CheckM provides only a very rough estimate of the actual community. 12 of 44 bins from *S. ocellatum* were for example classified as “bacteria” and the best taxonomic resolution is—with one exception (*Chlorogloea* bin32)—on order level (Table S2). Similarly, the authentic *C. purpurea* bin02 was classified only on phylum level as a cyanobacterium irrespectively of the excellent binning result (99.56% completeness, 0.29% contamination, 622× coverage), which are comparable to those of axenic cultures [33]. To gain a better taxonomic resolution, we investigated and compared the outcome of three different BLAST pipelines based on (i) the 16S-rRNA gene, (ii) the RpoB protein and (iii) a novel text mining approach for all 116 bins from the three cyanobacterial metagenomes (see Supplemental Text File S2A–C). The strengths and weaknesses of the three methods are shown in Table 3. It is remarkable that the 16S-rRNA gene, which usually serves as ultimate marker for a rapid taxonomic assessment, was not suitable for a reliable classification of metagenomic bins (Table S2, Text File S2A). The reason is that the 16S-rRNA genes have deviant nucleotide compositions and that many genomes comprise multiple copies of the rRNA operon [20]. The β subunit of the DNA-dependent RNA polymerase (RpoB), which was already the marker of choice for a rapid taxonomic analysis of genome-sequenced cyanobacteria [33], is also well-suited for MAGs (Table S3, Text File S2B). The comparably large RpoB protein with at least 1100 amino acid positions provides a good taxonomic resolution. It is essential, encoded by a single copy gene and should be diagnostic for the respective host bacterium. A consensus threshold for the species delineation is yet missing, but our analyses suggested that all strains with an RpoB amino acid identity of at least 96% belong to the same species. Finally, our text mining approach holds several advantages in comparison to the classifications based on a single marker (Table S4, supplemental Text File S2C). It is rapid, applicable for incomplete and contaminated bins and it allowed us to detect the 394 kb “plasmid” bin01 of *C. purpurea* with a higher coverage. Furthermore, it also provides taxonomic information for eukaryotic bins that might have otherwise been overlooked.

### 3.7. Microbial Composition of the Cyanosphere from S. ocellatum DSM 106950, C. purpurea SAG 13.99 and G. aponina DSM 107014

#### 3.7.1. Taxonomic Affiliation of Metagenomic Bins

**Figure 3:**
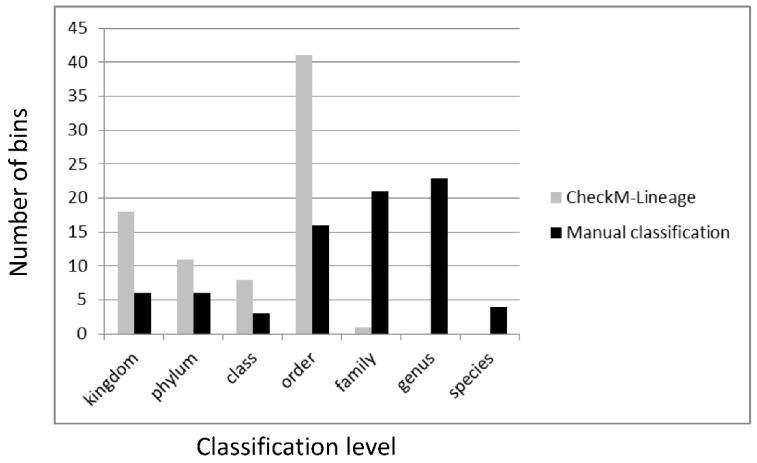
Taxonomic classification of 79 bins with a completeness of >80% obtained from three cyanobacterial metagenomes (Table S5). Gray columns show the automated classification by CheckM, black columns document the respective level after manual classification (RpoB, text mining).

The current study revealed a great microbial diversity in cultures of non-axenic cyanobacteria with up to 45 metagenomic bins. Key results from all bins that exhibit a more than 25-fold coverage and a completeness of at least 80% are shown in Table 4. Initially, a third of the bins were only characterized on kingdom level by CheckM (Table S5), this could be largely improved by our combination of 16S-rRNA, RpoB and text mining-based BLAST analyses (Table S5). CheckM retrieved the marker lineage “Bacteria” for incomplete and contaminated bins especially for poorly represented phyla such as *Chloroflexi* (*S. ocellatum*: bin34) and *Planctomycetes* or *Verrucomicrobia* (*S. ocellatum*: bin29; *C. purpurea*: bin07, bin21, bin24) even though they are nearly complete high-quality bins. However, our combined approach could classify them up to the family level (Table S5). This improvement in taxonomic classification, which allowed determining 28% and even 32% of the bins on the family and the genus level, respectively, is shown in Figure 3. Our rather conservative classification is balanced between overinterpretation and resolution, but the RpoB analyses resulted in a classification of 37 bins on species level (Table S3). Four of six bins that could only be classified on the kingdom level as “Bacteria” are characterized by a high contamination degree and low coverage or a missing RpoB sequence. The two remaining bins contain RpoB sequences with comparably low protein identities to the closest genome-sequenced relative (*C. purpurea*: bin15 [81.09%], *G. aponina*: bin14 [75.22%]; Table S3) and might represent first members of non- or under-sampled taxonomic lineages. Furthermore, the presence of at least one new family and 13 new genera that was proposed by our 16S-rDNA data (Table S2) does per se only allow a classification of the uncultured bacteria on order and family level, respectively. Accordingly, the combination of 16S-rDNA, RpoB and text mining provides a very good taxonomic resolution of low complexity metagenomes established via Illumina sequencing.

#### 3.7.2. Comparison of the Cyanobacterial Metagenomes

**Figure 4:**
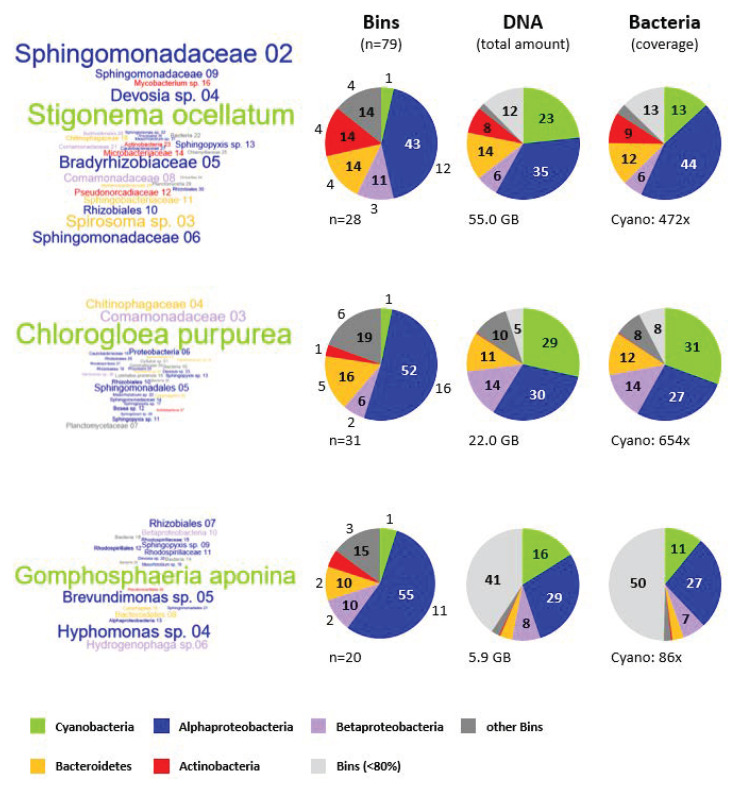
Relative abundance of bacterial bins in the cyanosphere of *Stigonema ocellatum* DSM 106950, *Chlorogloea purpurea* SAG 13.99 and *Gomphosphaeria aponina* DSM 107014. (**A**) Word clouds of metagenomic bins with a completeness of >80% (Table S5). The size of the taxon names corresponds to the genome coverage of the respective bins. The following color code was used to distinguish between the most abundant lineages on phylum/class level: green—*Cyanobacteria*, blue—*Alphaproteobacteria*, purple—*Betaproteobacteria*, orange—*Bacteroidetes*, red—*Actinobacteria*, dark gray—other bins, light gray—bins with a coverage below 80%. (**B**) Pie charts displaying the proportion of the bacterial lineages in percent. (i) The left pie charts show the relative abundance of the bins. The total number of bins per pie slice is also indicated. (ii) The middle pie charts show the total amount of DNA per bacterial lineage. Metagenome sizes range from 5.9 to 55.0 billion base pairs (Gbp). (iii) The right pie charts show the relative genome coverage of each category. The total genome coverage of the pivotal cyanobacterium is also shown.

The word clouds in Figure 4 visualize the taxonomic affiliation and coverage of all 79 bins with a completeness >80% by their color and size, respectively. The comparison documents the central position of the cyanobacterium feeding a zoo of associated heterotrophic microbes (see also Figure 1). The composition of *C. purpurea*’s cyanosphere exemplifies the outstanding role of the phototroph in comparison to the associated bacteria. The cyanobacterial communities of all three freshwater strains are strikingly dominated by *α*-, and *Betaproteobacteria* that are highlighted in blue and violet, respectively, followed by *Bacteroidetes* (yellow) and *Actinobacteria* (red). The left pie charts show that *Alphaproteobacteria* represent with about 50% of the bins the by far most abundant lineage of associated microbes in these consortia. *Rhizobiales* (16 bins) and *Sphingomonadales* (13 bins) are the typical alphaproteobacterial orders of the cyanosphere, in contrast to *Rhodospirillales* (4 bins), *Caulobacterales* (3 bins) and *Rhodobacterales* (1 bin) that occur only sporadically (Table 3, Table S5). The most abundant bin02 of the metagenome of *S. ocellatum* representing a *Sphingomonadaceae* exhibits with its conspicuous 472-fold genome coverage nearly a phototroph-heterotroph ratio of 1:1 (Figure 4). *Devosia* sp. bin04 and *Bradyrhizobiaceae* bin05 are the most abundant *Rhizobiales* that still comprise a genome equivalent proportion of more than 50% in comparison with *Stigonema*. *Sphingomonadales, Rhizobiales* and some other bacterial lineages have previously been identified as dominant players for the mineralization of cyanobacteria-derived particulate organic matter from a freshwater *Microcystis* bloom in the Chinese Lake Taihu [48]. Furthermore, our metagenome analysis identified seven betaproteobacterial bins with a completeness > 80% that are highlighted in violet (Figure 4). Five of them were classified as *Comamonadaceae* (*Burkholderiales*, Table S5), which is in agreement with a former study that showed the prominent role of this family among microbiomes of *Synechococcus*-dominated Nile River water samples [49]. One example is the complete RpoB sequence of the high-quality *G. aponina* bin06 (99.53% completeness, 1.40% contamination, 34× coverage) that obtained the best BLASTP hit *Hydrogenophaga* sp. 2FB (*Comamonadaceae*, WP_137922447.1; Table S2), which is a very close relative of the *Hydrogenophaga* bin1 from *Nostoc* strain ULC146 established in the pioneer metagenome study of non-axenic cyanobacteria [32]. Two abundant *Bacteroidetes* bins that are highlighted in yellow represent the second most common associated heterotrophs of *Stigonema* (*Spirosoma* sp. bin03; 234× cov.) and *Chlorogloea* (*Chitinophagaceae* bin04; 230× cov.), which reflects a successful niching strategy in both microbial communities (Figure 4). *Actinobacteria*, which are shown in red, are part of the non-dominant accompanying bacterial flora in the three investigated metagenomes. However, the metagenome of *S. ocellatum* that was sequenced to an exceptional depth of 55.0 billion base pairs resulted in three nearly complete genomes with a very low contamination level (*Pseudonocardiaceae* bin12 [99.17% compl.; 2.73% cont.; 106× cov.]; *Mycobacterium* sp. bin16 [100.00%; 0.13%; 74×]; *Actinobacteria* bin23 [95.26%; 0.00%; 41×]). It is noteworthy that promising bacteria with a unique phylogenetic position or novel biosynthesis gene clusters can due to the long-term stability of the microbial communities be isolated a posteriori. This is in comparison to metagenomic bins from environmental samples an invaluable benefit that reflects the hidden potential and great value of non-axenic bioresources from culture collections.

Beyond the numeric diversity of distinct bins in the three cyanobacterial metagenomes, we also compared the proportion of DNA of the five most abundant lineages. The middle pie charts in Figure 4 show that each cyanobacterial bin represents between 16% and 29% of the genetic information of the respective metagenome. This finding in combination with the observed coverages (see below) provides a guideline to calculate the sequencing depth of future metagenome projects that are just aimed to establish the genome sequence of the non-axenic cyanobacterium. The comparison of the left and the middle pie chart shows a reduction of the proportion of blue *Alphaproteobacteria*, which reflects the presence of many alphaproteobacterial bins with a comparably low coverage compared to those of the cyanobacterium. The middle pie chart comprises an additional light gray category showing the total DNA amount from all remaining bins that exhibit a coverage below 80%. Its low proportion of 5% in the *C. purpurea* and 12% in the *S. ocellatum* metagenome reflects a minor relevance for the total DNA composition. A different observation was made for the *G. aponina* metagenome comprising 41% of its total DNA amount in the light gray category of incomplete bins. This outcome does obviously not result from the comparably low sequencing depth of *Gomphosphaeria* (5.9 Gbp) in comparison with *Chlorogloea* (22.0 Gbp) and *Stigonema* (55.0 Gbp), because the proportion of all bins with a completeness below 80% is comparably low (Table S5). A plausible explanation is—analogous to the “root” bin comprising plasmid DNA of the cyanobacterium (see above)—a binning problem with the two most abundant, but only partial alphaproteobacterial bins of the *Gomphosphaeria* metagenome (bin01: 73.04% completeness, 216× coverage; bin02: 44.06%, 152×). Both bins might reflect variations in DNA composition of a single strain with a multipartite genome organization, which would accordingly represent an overlooked *Rhodospirillaceae* bacterium dominating the metagenome of *G. aponina* with an about 200-fold coverage.

The comparison of the coverage information shown in the right pie charts of Figure 4 provides, apart from the taxonomical composition (left) and the DNA content of the metagenome (middle), insights into the proportion of cyanobacteria in the low complexity communities. The diagram takes into account the wide range of genome sizes from less than 3 to more than 10 MB (Table S5). Accordingly, the fraction of *Stigonema* drops from 23% DNA amount (middle) to a coverage portion of 13% (right), representing genuine cyanobacterial genome equivalents in the non-axenic culture. A comparable value of 11% was observed for *G. aponina*. In contrast, *C. purpurea* accounts in agreement with the dominant word cloud for nearly a third of the total coverage (31%). Under the premise of (i) a comparable DNA preparation efficiency and (ii) an equal amount of genome equivalents per cell for all investigated bacteria, we conclude that the ratio of phototrophic to heterotrophic bacteria varies in the three investigated non-axenic cultures between 1:3 and 1:9. A single cyanobacterium does hence release sufficient amounts of metabolites to feed up to nine associated bacteria. This conclusion is in agreement with co-cultivation experiments of the marine cyanobacterium *Synechococcus* sp. WH7803 with *Ruegeria pomeroyi* that reached a stable 1:10 cell density equilibrium [7]. Comparable cell ratios were also reported for natural communities in the ocean [50], which provides independent evidence for the reliability of our indirect DNA-based calculation regarding the organismal composition of the cyanosphere.

### 3.8. Eukaryotic Contaminations in the Cyanosphere

A rather unexpected finding was the identification of nine partial eukaryotic RpoB sequences in two “archaeal” bins of *S. ocellatum* (bin40, bin41; Table S3). The 2.5-fold coverage of both bins is just above the detection limit, but it exemplifies that deep Illumina sequencing of non-axenic cyanobacteria allows the identification of authentic eukaryotic contaminations. Two small RpoB fragments of 283 aa and 181 aa showed *Basidiobolus meristosporus* CBS 931.73 (ORY01079.1) as best BLASTP hit, which indicates that at least one fungus lives in association with the filamentous cyanobacterium *Stigonema*. However, the fungal hits exhibit only a low sequence identity between 50% and 74% thus documenting that their actual taxonomic affiliation is unclear. Furthermore, three partial RpoB sequences of 468 aa, 402 aa and 80 aa had *Acanthamoeba castellanii* strain Neff (XP_004348530.1) as best hit, which is compatible with the visual detection of associated amoeba via light microscopy. The fragmented RpoB sequences are insufficient for a taxonomic classification of the amoeba in the *Stigonema* metagenome, but the 16S dataset also comprises a partial eukaryotic 18S-rDNA sequence of 936 bp that shows a similarity of 97% with *Mycamoeba gemmipara* (*Discosea*, *Flabellinia*, *Mycamoeba*; KX687875.1; Table S1). This affiliation clearly documents that the amoeba belongs to the family *Dermamoebidae* [51]. The amoeba is probably grazing the abundant heterotrophic bacteria in the cyanosphere of *Stigonema* (Figure 1A) thus indicating the presence of a simple food chain in our test tube.

The metagenome of *Chlorogloea* lacks any RpoB hint of eukaryotic contaminations, but it also comprises a partial 18S-rDNA sequence of 818 bp exhibiting 100% sequence identity with the *Platyamoeba* strain VV/I (*Discosea*, *Flabellinia*, *Vannellidae*; AY929923.1), which was isolated from tap water in Germany [52]. A comparable observation was made for the *Gomphosphaeria* metagenome that is lacking eukaryotic RpoB sequences, but showed two amoebal hits in the 16S dataset that might belong to the same organism (Table S2). Bin16 comprises a 778 bp fragment of the mitochondrial 16S-like rDNA gene, representing one of the first molecular markers for the investigation of protist evolution [53], that exhibits a comparably low sequence similarity of 78% with the heterolobosean amoeba *Naegleria jadini* (AY376154.1). The genuine eukaryotic 18S rDNA sequence of 983 bp shows a sequence similarity of 99.9% with the thermophilic representative *Fumarolamoeba ceborucoi* strain FUM1 (*Heterolobosea*, *Vahlkamphiidae*; FR719836.1) that has been isolated near a fumarole at a volcano in Mexico [54]. The origin of the cyanobacterial host *G. aponina*, which was isolated from the Neusiedlersee in Austria, indicates that the associated amoeba is a temperate representative of the species *F. ceborucoi*. Taken together, the in silico detection of discosean or heterolobosan amoeba in all three investigated cyanobacterial metagenomes confirms our initial observations based on light microscopy.

### 3.9. Phylogenetic Analyses of 213 Cyanobacterial Genomes

One aim of the current study was to add missing branches to the cyanobacterial tree of life. Our rationale for the taxon sampling in 2018 was a distinct relatedness of cyanobacterial strains, which were deposited in the public culture collections of the DSMZ and the SAG, to genome-sequenced relatives of the NCBI database. Due to the lack of a general taxonomic classification scheme of cyanobacteria that is based on their evolutionary relationships, we choose the NCBI taxonomy as a proxy for our comparison. The highest taxonomic rank of cultured cyanobacteria without available reference genome sequences was the family level. Accordingly, the establishment of high-quality MAGs of *S. ocellatum* DSM 106950 (*Stigonemataceae*), *C. purpurea* SAG 13.99 (*Entophysalidaceae*) and *G. aponina* DSM 107014 (*Gomphosphaeriaceae*) paved the way to determine the phylogenetic position of three new families in the cyanobacterial tree.

**Figure 5:**
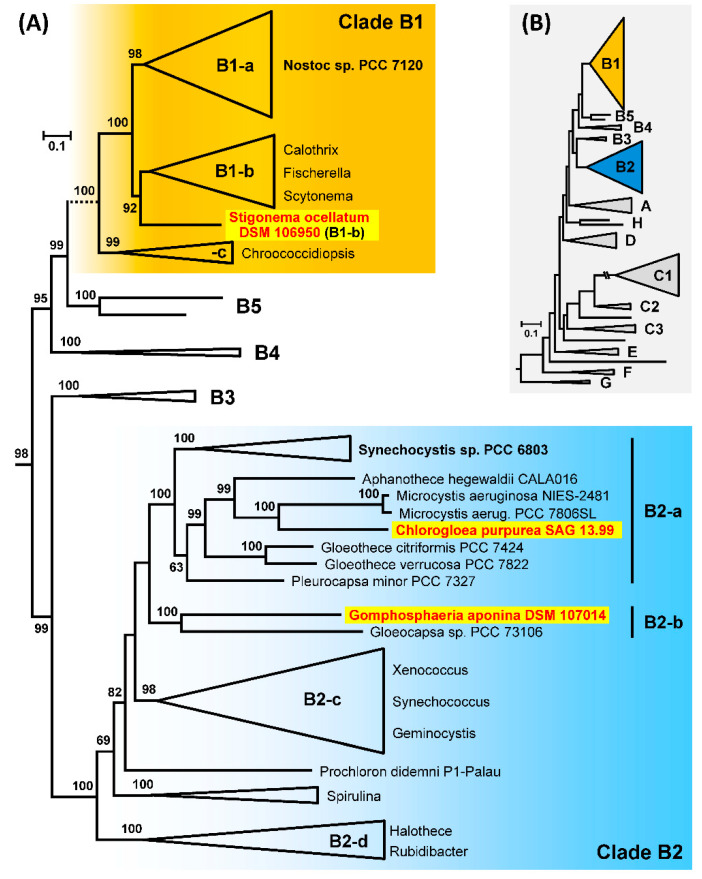
Phylogenetic maximum likelihood tree of 213 genome-sequenced cyanobacteria. (**A**) The MLSA phylogeny was inferred by RAxML under the GTR + 4Γ model based on a concatenated alignment of 43 proteins with 6458 amino acid positions. Bootstrap support of 100 replicates is indicated. The branching pattern of clade B1 corresponds to a subanalysis of 70 strains (Figure S3). Three cyanobacteria investigated in the current study are highlighted in red with a yellow background. (**B**) Schematic overview of the cyanobacterial diversity (see MLSA tree of 213 taxa; Figure S2). The cyanobacterial clades were designated according to Shih et al. (2013). Their division in different subclades (e.g., B1-a, -b, -c) is based on the current analysis.

A comprehensive MLSA phylogeny of the three established MAGs and 210 cyanobacterial reference genomes, which largely corresponds to the taxon sampling of Will et al. (2019) [33], is shown in Figure S1. The tree comprises eight distinct major branches, designated clade A to clade H, that represent the phylogenetic backbone of cyanobacteria and were essentially described in the first diversity-driven genome study of this phylum [55]. *S. ocellatum* belonging to the order *Nostocales* is located in subclade B1, whereas *C. purpurea* and *G. aponina* that are both representing the order *Chroococcales* are placed in subclade B2 (Figure 5). All three strains show a distinct phylogenetic position, which is in agreement with their proposed status as the first genome-sequenced representative of a cyanobacterial family. First, the MLSA tree clearly shows the common branching of *C. purpurea* SAG 13.99 and the toxic bloom-forming cyanobacterium *Microcystis aeruginosa*, which is eponymous for the “Fast-Death Factor” microcystin [56]. The next relative is *Aphanothece hegewaldii* followed by two *Gloeothece* strains, which are all located in subclade B2-a that also contains the model organism *Synechocystis* sp. PCC 6803 (Figure 5). However, it remains unclear if *C. purpurea* SAG 13.99 or *Chlorogloea* sp. CCALA 695, which is located in subclade B1-c (Figure S2), is a genuine representative of the genus *Chlorogloea* (see above; Figure S2, Table S6). The polyphyly of the two strains challenges the general classification of *Entophysalidaceae*. Second, the phylogenetic tree with 213 strains documents the common branching of *G. aponina* (*Gomphosphaeriaceae*) together with *Gloeocapsa* sp. PCC 73106 (*Chroococcaceae*) that is despite of the early separation of the two taxa supported by 100% bootstrap proportion (BP). Both cyanobacteria are located in subclade B2-b. Third, the comprehensive MLSA analysis clearly showed in agreement with former 16S rDNA analyses [57] a phylogenetic localization of *S. ocellatum* in clade B1 (Figure S2), but its actual position was only poorly supported. A nested position among nostocalean cyanobacteria was recently proposed by comparative analyses of heterocyte glycolipids that indicated a close relationship of *S. ocellatum* SAG 48.90 (= DSM 106950) with *Rivulariaceae* and *Scytonemataceae* [58]. A subanalysis of the 70 taxa of clade B1 improved the phylogenetic resolution and supports the placement of *Stigonema* within subclade B1-b with 92% BP (Figure S3, Figure 5). The proposed sister position of *S. ocellatum* to a branch with three subtrees mainly comprising taxa of the genera *Calothrix*, *Fischerella* and *Scytonema* has to be validated by future phylogenomic analyses.

## 4. Discussion

### 4.1. Naming and Classification of Cyanobacteria

The current nomenclature of cyanobacteria reflects the historical and still unresolved dilemma between the “botanical” and the “prokaryotic” code [10,59]. Their global taxonomy is rather static and in contrast to other bacterial lineages, whose taxonomic classification is continuously improved by phylogenetic analyses of their type strain genomes [60,61], reminiscent of a “frozen accident”. The species names of cyanobacteria from culture collections such as the French Pasteur Culture collection (PCC), the Japanese NIES collection or the German DSMZ usually reflect the original designation of the depositor if no apparent misclassification was observed. However, the phylogenetic trees that were established in this study provide a solid basis to assess the current classification of cyanobacteria. The NCBI-based taxonomy of all 213 investigated strains is shown in Table S6, its sequential ordering from *Nostoc* sp. PCC 7120 to *Gloeobacter kilaueensis* JS1 is corresponding to the phylogeny in Figure S2. Their classification on family and order level is largely consistent with those of the Czech on-line database of cyanobacterial genera, CyanoDB 2.0, which was developed as a reference tool for taxonomists based on primary species descriptions and molecular data [35]. However, the comparison between the NCBI taxonomy and the CyanoDB classification showed that about 10% of the strains (20/213) were placed into different families, four strain names represent invalid taxa, whose nomenclature does not meet the ICN/ICNP rules for holotypes/type strains, and the three remaining ones that are represented by two *Moorea producens* strains and *Dactylococcopsis salina* PCC 8305 obtained no results (Table S6). The manual curation of taxonomic data by Jiří Komárek and Tomáš Hauer led to an improvement in cyanobacterial classification, which is exemplified by an assessment of the genera *Sphaerosphermopsis*, *Raphidiopsis*, *Cuspidothrix* and *Nodularia*—are all located in subclade B1-a (Figure S2)—as *Nostocaceae*. Accordingly, the classification by CyanoDB was used as a reference for our further taxonomic considerations.

### 4.2. Previous Classification of the Genera Stigonema, Gomphosphaeria and Chlorogloea

Among the diversity of filamentous strains, *Stigonema* served in the past as the type genus for true-branching cyanobacteria of Subsection V, whereas no or false-branching cyanobacteria of Subsection IV were represented by the genus *Nostoc* [6]. However, the polyphyly of the “*Stigonematales*” as well as a nested positioning of *S. ocellatum* within the order *Nostocales* has previously been shown by phylogenetic and biochemical analyses [57,58].

*Gomphosphaeria* was reported as one of the dominant cyanobacterial genera in Irish lakes [62], but virtually no strains are deposited in public culture collections and only a single nucleotide sequence was yet deposited at GenBank. The respective 16S rRNA gene of *G. aponina* (KM019999) exhibits a sequence identity of less than 94% with the next cultivated relative, which reflects its distinct positioning in the cyanobacterial tree of life.

*Chlorogloea* represents a poorly studied cyanobacterial genus and the morphology-based classification of several cultivated strains is contradictory to their phylogenetic affiliation. The 16S rRNA gene of *C. purpurea* SAG 13.99 (KM019990.1) shows a specific association with *M. aeruginosa* located in subclade B2 of the cyanobacterial species tree [55]. This position is in conflict with the close relationship of the genome-sequenced strain *Chlorogloea* sp. CCALA 695 grouping together with *Synechocystis* sp. PCC 7509 in clade B1 [33]. Furthermore, neither *C. microcystoides* SAG 10.99 (KM019955.1) nor two Brasilian *Chlorogloea* isolates (CENA150, CENA152 [63]) exhibit a specific affiliation with the two strains mentioned above, which documents the polyphyly of *Chlorogloea* isolates located in at least four different subclades of the cyanobacterial species tree. Our comparison hence reflects the limits of a reliable classification of unicellular cyanobacteria according to morphological criteria [10].

### 4.3. Genome-Derived Phylogenies Show Incongruencies in Cyanobacterial Taxonomy

Metagenome sequencing of *S. ocellatum* DSM 106950, *G. aponina* DSM 107014 and *C. purpurea* SAG 13.99 was the basis to determine their phylogenetic position and to fill some gaps in the cyanobacterial tree of life. Our phylogenetic analyses allowed us to pinpoint some taxonomic incongruencies of the investigated cyanobacteria. *Chlorogloea*, *Synechocystis* and *Synechococcus* are predestined genera for taxonomy-based pitfalls resulting from the sampling of allegedly closely related strains. Metagenome sequencing allowed a precise positioning of *C. purpurea* SAG 13.99 in subclade B2-a of the cyanobacterial species tree (Figure 5), which is incongruent with the placement of *Chlorogloea* sp. CCALA 695 in subclade B1-c (Figure S2). The polyphyly of unicellular *Chlorogloea* strains documents the need for a meaningful reclassification beyond morphological criteria (see also above). A second example is *Synechocystis* sp. PCC 6714 that groups together with the model organism *Synechocystis* sp. PCC 6803 in subclade B2-a, while *Synechocystis* sp. PCC 7509 has a very distant position in subclade B1-c (Figure S2). Third, *Synechococcus* strains are even present in at least nine distinct clades of the cyanobacterial tree, i.e., B2-c (PCC 7117, PCC 7002), C1-a (WH 7803, WH 8102), C1-b (WH 5701), C1-c (RCC307), C2 (PCC 7942), C3 (PCC 7335), E (PCC 6312), F (PCC 7502) and G (PCC 7336). In a first genome-based study Coutinho et al. proposed to classify the *Synechococcus* strains of clade C1-a, representing the sister lineage of the genus *Prochlorococcus* (Figure S2), as *Parasynechococcus* [64]. However, this new genus has never been validated, probably due to formal reasons resulting from the incompatibility of the botanical and prokaryotic code [10]. In a very recent comparative genome study the taxonomic dilemma was summarized as follows “*Although there is no doubt that the organisms classified as Synechococcus are polyphyletic, there is not yet a consensus on how to classify them*” [65].

The taxonomic problems exemplified above document the need for a better and consistent classification of cyanobacteria. A reconciliation of the different codes of nomenclature (ICN, “Botanical Code”; ICNP, “Prokaryotic Code”) is needed to overcome the apparent standoff. A description of neotypes and their deposition in public culture collections would solve the problem of lacking reference strains for morphological, physiological and molecular comparisons. This proceeding should ideally be guided by the establishment of genome data from the original-type material deposited in herbaria.

## 5. Conclusions

16S rDNA and metagenome analyses of environmental samples provided astonishing insights into the microbial diversity of our planet. The current study shows that non-axenic cyanobacteria represent promising resources to fill the gaps in the cyanobacterial tree of life and to investigate the hidden potential of low complexity communities. The ultimate advantage of culture-dependent metagenomics is a retrospective access to microbes that stably coexisted with the cyanobacterial host for decades. Metagenome binning of three distantly related cyanobacteria from individual freshwater habitats allowed us to retrace the microbial composition of the cyanosphere (Table 4). The communities comprise up to 40 associated heterotrophs and they are dominated by *Alphaproteobacteria* and *Bacteroidetes* (Figure 4). The detection of at least one new family and about a dozen uncharacterized bacterial genera in the investigated consortia document that part of the microbial “dark matter” is already cultivated.

High-throughput Illumina sequencing on the NovaSeq platform was the basis to establish large amounts of genome data from three fastidious cyanobacteria. Metagenomic binning in combination with manual curation resulted in cyanobacterial genomes of highest quality, whose completeness and contamination-level is comparable to those of axenic strains [33]. The major challenge of the current study was the reliable taxonomic classification of metagenomic bins, which was essentially ensured by the diagnostic marker RpoB and a novel text mining approach. The binning problems of the 16S-rDNA reflect the limits of Illumina short-read sequencing, but recent technical achievements in long-read sequencing provide promising perspectives to overcome these obstacles soon.

## Figures and Tables

**Table 1:** Morphologic, genetic and biogeographic characteristics of three freshwater cyanobacteria.

	*Stigonema ocellatum*	*Chlorogloea purpurea*	*Gomphosphaeria aponina*
**Morphology**			
Cells	filamentous	unicellular	unicellular-colonial
Cell shape	uniseriate	spherical	heart-shaped cells
Sheath	+	+	+
Branching	T-branching	-	-
Heterocysts	intercalary	-	-
**Phylogeny**			
Clade ^1^	B1-b	B2-a	B2-b
Order	*Nostocales*	*Chroococcales*	*Chroococcales*
Family	*Stigonemataceae*	*Entophysalidaceae*	*Gomphosphaeriaceae*
**Genome**			
MAG status	high-quality draft	high-quality draft ^2^	high-quality draft ^2^
Contigs	509	228	749
Size (Mb)	10.35	4.74	5.34
G + C	43.8	45.3	38.9
Accession number	JADQBA000000000	JADQBB000000000	JADQBC000000000
**Isolation**			
Origin	Allgäu, Germany	Serra da Estrela, Portugal	Neusiedlersee, Austria
Habitat	freshwater	freshwater	freshwater
Locality	*Sphagnum* bog	flowing water	benthic on *Phragmites*
Scientist	D. Mollenhauer	M. F. Santos	E. Kusel-Fetzmann
Year	1970	1981	1985
Publications	Gugger and Hoffmann (2004) Bauersachs et al. (2019)	-	-
**Collection ID**			
DSM	106950	-	107014
SAG	48.90	13.99	52.96

^1^ Shih et al. (2013) and current study; ^2^ 5S-rRNA gene is missing; + present; - absent.

**Table 2:** Properties and statistics for metagenome-assembled genomes of three non-axenic cyanobacteria. The MAGs are deposited at the NCBI under the BioProject accession: PRJNA659938.

Attribute	*Stigonema ocellatum* DSM 106950		*Chlorogloea purpurea* SAG 13.99		*Gomphosphaeria aponina* DSM 107014	
	Value	% of Total	Value	% of Total	Value	% of Total
Genome size (bp)	10,354,468	100.00	4,737,903	100.00	5,337,155	100.00
DNA coding (bp)	7,983,295	77.10	4,140,927	87.40	4,360,456	81.70
DNA G + C (bp)	4,535,257	43.80	2,146,270	45.30	2,081,490	39.00
Contigs	509		228		749	
Total genes	8824	100.00	4429	100.00	5305	100.00
Protein coding genes	8701	98.61	4384	98.98	5235	98.68
RNA genes	3	0.03	2	0.05	2	0.04
Pseudo genes	120	1.36	43	0.97	68	1.28
Genes with function prediction	2826	32.03	1931	43.60	1846	34.80
Genes assigned to COGs	4011	45.46	2250	50.80	2413	45.49
Genes with Pfam domains	5985	67.83	3249	73.36	3751	70.71
Genes with signal peptides	664	7.52	350	7.90	446	8.41
Genes with transmembrane helices (≥3)	708	8.02	449	10.14	378	7.13
CRISPR repeats	13	0.15	10	0.23	19	0.36

**Table 3:** Comparison of 16S-rRNA, RpoB and text mining approaches for the classification of metagenomic bins.

**16S-rRNA Gene** (BLASTN)	
**Pro**	**Contra**
(1) Gold standard for prokaryotic taxonomy	(1) Frequent lack of marker gene in the bins *
(2) Well-curated reference sequences from all type strains	(2) Wrong marker gene(s) in the bins *
(3) Large set of reference sequences (isolates, environment)	(3) Comparably poor taxonomic resolution
(4) Thresholds for delineation of species and higher order taxa	
**RpoB Protein** (BLASTP)	
**Pro**	**Contra**
(1) Representative codon usage and nucleotide composition (essential protein)	(1) No general thresholds for species delineation
(2) Coverage diagnostic for the genome (single-copy gene)	(2) Inconclusive classification of bins with a high contamination level
(3) Large protein with good taxonomic resolution	
(4) Present in most bins with a completeness > 90%	
**Text Mining** (BLASTN)	
**Pro**	**Contra**
(1) Rapid assessment of metagenomes	(1) Taxonomic resolution limited to genus level
(2) Applicable for incomplete and contaminated bins	
(3) Reliable identification of the dominant (primary) genome	
(4) Detection of plasmid-related bins lacking any marker gene	
(5) Identification of eukaryotic bins	

* binning problems based on a deviant nucleotide composition and copy number of the 16S-rRNA gene.

**Table 4:** Metagenomic binning of three non-axenic cyanobacteria and taxonomic classification. 36 bins with a completeness of >80% and a coverage >25-fold are shown. Cyanobacterial bins are highlighted in bold and gray.

**(A) *Stigonema ocellatum* DSM 106950**										
**Binning Results**						**Classification ^1^**				
**bin**	**Compl.**	**Contam.**	**Contigs**	**Genome (bp)**	**Coverage**	**16S**	**RpoB**	**Text**	**RpoB & Text Mining**	**Tax.**
**01**	**99.28**	**2.35**	**515**	**10,519,008**	**471.84**	**yes/no**	**“yes”**	**“yes”**	**S. ocellatum ^2^**	**s**
02	96.00	3.33	106	3,017,240	415.54	no	yes	yes	Sphingomonadaceae	f
03	100.0	0.74	184	7,762,495	244.90	false	yes	yes	Spirosoma sp.	g
04	80.80	5.34	36	4,445,593	244.15	x	yes	yes	Devosia sp.	g
05	86.21	33.89	69	4,652,777	242.25	no	yes	yes	Bradyrhizobiaceae	f
06	99.59	8.90	28	4,785,235	219.72	x	yes	yes	Sphingomonadaceae	f
08	98.44	2.23	36	6,359,601	149.04	x	x	yes	Comamonadaceae	f
09	93.10	36.99	134	7,299,278	143.94	x	yes/no	mult.	Sphingomonadaceae	f
10	83.92	3.82	94	8,160,230	142.77	x	yes	yes	Rhizobiales	o
11	98.10	0.71	62	4,257,773	122.35	false	yes	yes/no	Sphingobacteriaceae	f
12	99.17	2.73	79	7,053,193	106.66	x	yes	yes	Pseudonorcadiaceae	f
13	99.66	0.63	53	3,680,574	98.53	x	yes	yes	Sphingopyxis sp.	g
14	97.81	0.27	28	2,624,238	94.72	yes	yes	yes	Microbacteriaceae	f
16	100.00	0.13	33	6,260,391	74.38	x	yes	yes	Mycobacterium sp.	g
18	99.51	1.08	49	6,025,211	61.24	yes	yes	yes/no	Chitinophagaceae	f
21	92.52	2.80	114	7,675,694	41.20	x	x	yes	Comamonadaceae	f
22	98.46	2.72	19	4,521,530	41.19	x	x	yes/no	Bacteria	k
23	95.26	0.00	148	3,446,731	41.02	yes	yes	yes	Actinobacteria	p
**(B) *Chlorogloea purpurea* SAG 13.99**										
**Binning Results**						**Classification ^1^**				
**bin**	**Compl.**	**Contam.**	**Contigs**	**Genome (bp)**	**Coverage**	**16S**	**RpoB**	**Text**	**RpoB & Text Mining**	**Tax.**
01 + 02 *	**100.00**	**0.29**	**186**	**4,595,485**	**654.42**	**x**	**“yes”**	**“Yes”**	**C. purpurea^2^**	**s**
03	85.86	5.70	131	4,964,628	288.68	x	x	yes	Comamonadaceae	f
04	97.04	0.52	37	4,500,700	230.28	yes	yes	yes	Chitinophagaceae	f
05	81.59	14.77	93	4,443,934	150.84	false	x	yes/no	Sphingomonadales	o
06	98.12	30.88	130	6,184,863	105.77	x	mult.	mult.	Proteobacteria	p
07	91.03	2.30	81	7,916,241	87.81	x	yes	yes/yes	Planctomycetaceae	f
10	89.39	25.52	79	8,815,014	60.85	x	x	yes/no	Rhizobiales	o
11	85.54	10.13	48	4,001,378	52.94	x	yes	yes	Sphingopyxis sp.	g
12	93.73	34.01	91	8,062,010	45.99	x	yes	yes	Bosea sp.	g
13	89.40	17.80	58	3,664,549	37.48	x	false	yes	Sphingopyxis sp.	g
14	99.09	1.75	36	3,405,585	31.73	yes	yes	yes	Sphingomonadaceae	f
15	97.62	0.98	33	4,008,507	28.58	no	yes	yes/no	Bacteria	k
16	100.0	13.79	72	5,989,586	25.63	yes	yes	yes/no	Luteitalea pratensis	s
**(C) *Gomphosphaeria aponina* DSM 107014**										
**Binning Results**						**Classification ^1^**				
**bin**	**Compl.**	**Contam.**	**Contigs**	**Genome (bp)**	**Coverage**	**16S**	**RpoB**	**Text**	**RpoB & Text Mining**	**Tax.**
**03**	**97.82**	**0.11**	**746**	**5,354,489**	**85.91**	**x**	**“yes”**	**“Yes”**	**G. aponina ^2^**	**s**
04	100.00	5.55	277	4,200,065	58.22	yes	yes	mult.	Hyphomonas sp.	g
05	94.48	4.22	247	2,796,749	49.08	yes/no	yes	yes	Brevundimonas sp.	g
06	99.53	1.40	378	4,670,292	34.15	x	yes	yes	Hydrogenophaga sp.	g
07	98.54	2.51	174	3,619,276	29.06	false	yes	yes	Rhizobiales	o

^1^ for details see Table S5; ^2^ reference strain; * manual curated dataset; Compl., completeness; Contam., contamination; Tax., taxonomic level of classification; k, kingdom; p, phylum; o, order; f, family; g, genus; s, species.

## Data Availability

The data presented in this study are openly available in the NCBI sequence database (BioProject: PRJNA659938; Sequence Read Archive [SRA]: SRR12487250, SRR12487251, SRR12487252).

## Supplementary Materials

The following are available online at https://www.mdpi.com/2073-4425/12/3/389/s1. Table S1: Comparison of the cyanobacterial MAGs obtained by MaxBin, Concoct and MetaBAT as well as their comparative assessment with DAS Tool. Table S2: Analysis of 16S-rDNA sequences from metagenomic binning and comparison with taxonomic marker lineage affiliation. Table S3: Analysis of RpoB protein sequences from metagenomic binning and comparison with taxonomic marker lineage affiliation. Table S4: Result of BLASTN-based taxonomy mining and comparison with taxonomic marker lineage affiliation. Table S5: Result of BLASTN-based taxonomy mining and comparison with taxonomic marker lineage affiliation. Table S6: Comparison of the phylogenetic affiliation of 213 Cyanobacteria with the NCBI taxonomy (see MLSA-tree, Figure S2). Figure S1: Scanning electron microscopy of three non-axenic cyanobacteria. Figure S2: MLSA-Phylogeny of 213 Genome-sequenced Cyanobacteria. Figure S3: MLSA-Phylogeny of 70 Genome-sequenced Cyanobacteria (Clade B1). Text Files S1: Text mining as analysis tool for metagenomes (Illumina). Text Files S2: Classification of metagenomic bins from non-axenic cyanobacteria.
