# Supplementary material for: Filling the Gaps in the Cyanobacterial Tree of Life—Metagenome Analysis of Stigonema ocellatum DSM 106950, Chlorogloea purpurea SAG 13.99 and Gomphosphaeria aponina DSM 107014
Source: Genes (Basel). 2021 Mar 9;12(3):389. doi: 10.3390/genes12030389 (PMC8001431; doi:10.3390/genes12030389)
Supplement: Supplementary file 1 [file genes-12-00389-s001.zip › genes-1023673-SI/B_Supplemental-Data_Revision_Marter-et-al-210203/FigureS1_EM-Microscopy_Cell-Types_210203.pptx]

## Slide 1
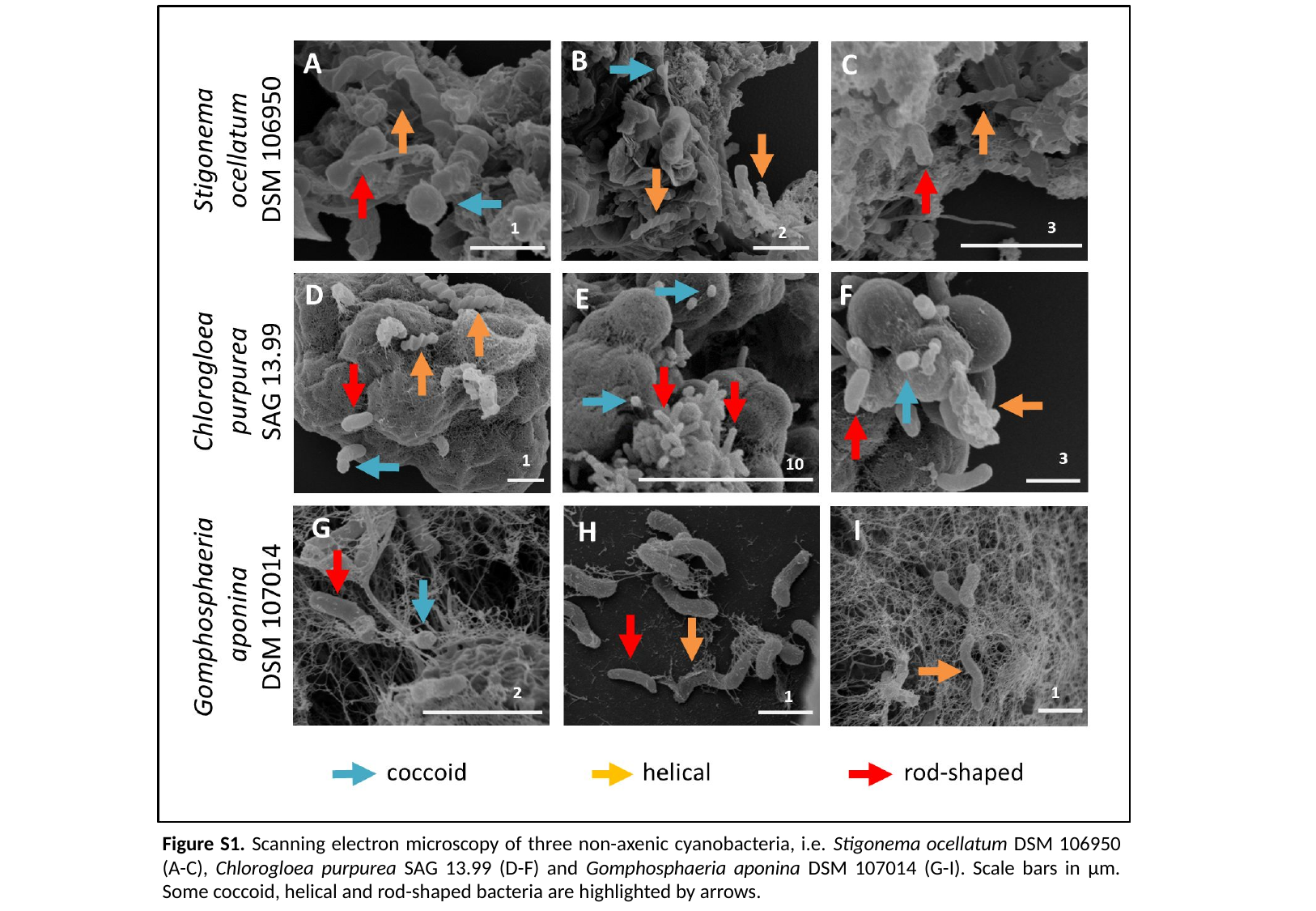

Figure S1. Scanning electron microscopy of three non-axenic cyanobacteria, i.e. Stigonema ocellatum DSM 106950 (A-C), Chlorogloea purpurea SAG 13.99 (D-F) and Gomphosphaeria aponina DSM 107014 (G-I). Scale bars in µm. Some coccoid, helical and rod-shaped bacteria are highlighted by arrows.
