# Supplementary material for: Filling the Gaps in the Cyanobacterial Tree of Life—Metagenome Analysis of Stigonema ocellatum DSM 106950, Chlorogloea purpurea SAG 13.99 and Gomphosphaeria aponina DSM 107014
Source: Genes (Basel). 2021 Mar 9;12(3):389. doi: 10.3390/genes12030389 (PMC8001431; doi:10.3390/genes12030389)
Supplement: Supplementary file 1 [file genes-12-00389-s001.zip › genes-1023673-SI/B_Supplemental-Data_Revision_Marter-et-al-210203/FigureS2_Cyano-213_CheckM_5_20_R213_V6011_RaxGTR4g_210129.pptx]

## Slide 1
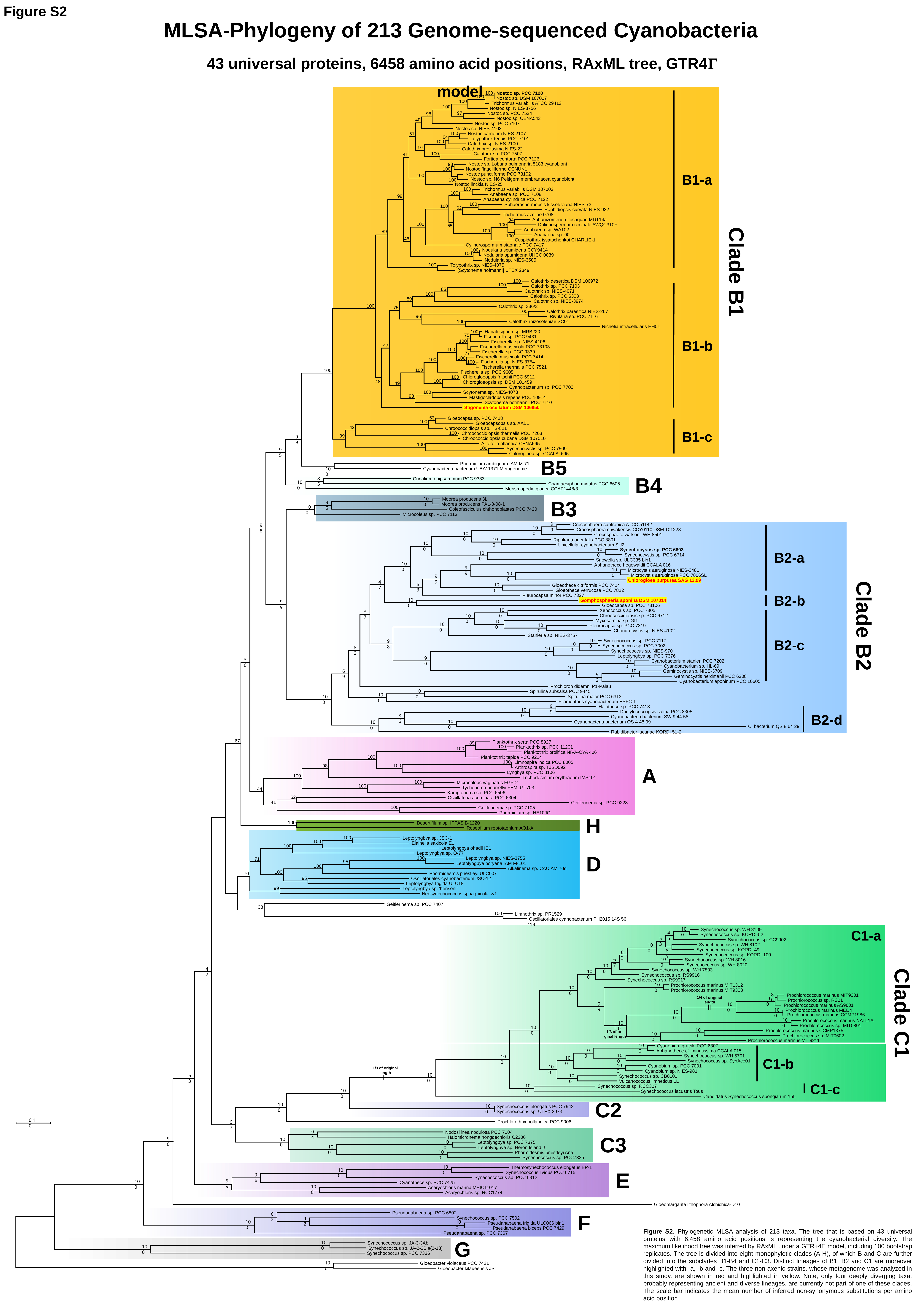

Figure S2
MLSA-Phylogeny of 213 Genome-sequenced Cyanobacteria
43 universal proteins, 6458 amino acid positions, RAxML tree, GTR4G model
B1-a
Clade B1
B1-b
B1-c
100
100
100
100
97
98
40
100
51
64
100
97
100
41
98
100
100
100
100
100
99
100
100
62
84
100
100
55
100
89
100
46
100
100
100
100
100
85
100
89
100
75
100
96
100
100
75
100
42
100
77
100
100
100
100
100
100
48
49
100
98
 Nostoc sp. PCC 7120
 Nostoc sp. DSM 107007
 Trichormus variabilis ATCC 29413
 Nostoc sp. NIES-3756
 Nostoc sp. PCC 7524
 Nostoc sp. CENA543
 Nostoc sp. PCC 7107
 Nostoc sp. NIES-4103
 Nostoc carneum NIES-2107
 Tolypothrix tenuis PCC 7101
 Calothrix sp. NIES-2100
 Calothrix brevissima NIES-22
 Calothrix sp. PCC 7507
 Fortiea contorta PCC 7126
 Nostoc sp. Lobaria pulmonaria 5183 cyanobiont
 Nostoc flagelliforme CCNUN1
 Nostoc punctiforme PCC 73102
 Nostoc sp. N6 Peltigera membranacea cyanobiont
 Nostoc linckia NIES-25
 Trichormus variabilis DSM 107003
 Anabaena sp. PCC 7108
 Anabaena cylindrica PCC 7122
 Sphaerospermopsis kisseleviana NIES-73
 Raphidiopsis curvata NIES-932
 Trichormus azollae 0708
 Aphanizomenon flosaquae MDT14a
 Dolichospermum circinale AWQC310F
 Anabaena sp. WA102
 Anabaena sp. 90
 Cuspidothrix issatschenkoi CHARLIE-1
 Cylindrospermum stagnale PCC 7417
 Nodularia spumigena CCY9414
 Nodularia spumigena UHCC 0039
 Nodularia sp. NIES-3585
 Tolypothrix sp. NIES-4075
 [Scytonema hofmanni] UTEX 2349
 Calothrix desertica DSM 106972
 Calothrix sp. PCC 7103
 Calothrix sp. NIES-4071
 Calothrix sp. PCC 6303
 Calothrix sp. NIES-3974
 Calothrix sp. 336/3
 Calothrix parasitica NIES-267
 Rivularia sp. PCC 7116
 Calothrix rhizosoleniae SC01
 Richelia intracellularis HH01
 Hapalosiphon sp. MRB220
 Fischerella sp. PCC 9431
 Fischerella sp. NIES-4106
 Fischerella muscicola PCC 73103
 Fischerella sp. PCC 9339
 Fischerella muscicola PCC 7414
 Fischerella sp. NIES-3754
 Fischerella thermalis PCC 7521
100
 Fischerella sp. PCC 9605
63
100
42
100
99
100
100
 Gloeocapsa sp. PCC 7428
 Gloeocapsopsis sp. AAB1
 Chroococcidiopsis sp. TS-821
 Chroococcidiopsis thermalis PCC 7203
 Chroococcidiopsis cubana DSM 107010
 Aliterella atlantica CENA595
 Synechocystis sp. PCC 7509
 Chlorogloea sp. CCALA 695
 Chlorogloeopsis fritschii PCC 6912
 Chlorogloeopsis sp. DSM 101459
 Cyanobacterium sp. PCC 7702
 Scytonema sp. NIES-4073
 Mastigocladopsis repens PCC 10914
 Scytonema hofmannii PCC 7110
 Stigonema ocellatum DSM 106950
99
100
 Phormidium ambiguum IAM M-71
 Cyanobacteria bacterium UBA11371 Metagenome
95
85
100
 Crinalium epipsammum PCC 9333
 Chamaesiphon minutus PCC 6605
 Merismopedia glauca CCAP1448/3
100
95
100
99
 Moorea producens 3L
 Moorea producens PAL-8-08-1
 Coleofasciculus chthonoplastes PCC 7420
 Microcoleus sp. PCC 7113
B5
B4
B3
99
100
100
100
100
100
100
100
99
100
100
99
47
63
100
100
100
37
100
100
100
100
100
98
100
82
100
99
100
69
92
100
100
100
99
100
86
100
100
 Crocosphaera subtropica ATCC 51142
 Crocosphaera chwakensis CCY0110 DSM 101228
 Crocosphaera watsonii WH 8501
 Rippkaea orientalis PCC 8801
 Unicellular cyanobacterium SU2
 Synechocystis sp. PCC 6803
 Synechocystis sp. PCC 6714
 Snowella sp. ULC335 bin1
 Aphanothece hegewaldii CCALA 016
 Microcystis aeruginosa NIES-2481
 Microcystis aeruginosa PCC 7806SL
 Chlorogloea purpurea SAG 13.99
 Gloeothece citriformis PCC 7424
 Gloeothece verrucosa PCC 7822
 Pleurocapsa minor PCC 7327
 Gomphosphaeria aponina DSM 107014
 Gloeocapsa sp. PCC 73106
 Xenococcus sp. PCC 7305
 Chroococcidiopsis sp. PCC 6712
 Myxosarcina sp. GI1
 Pleurocapsa sp. PCC 7319
 Chondrocystis sp. NIES-4102
 Stanieria sp. NIES-3757
 Synechococcus sp. PCC 7117
 Synechococcus sp. PCC 7002
 Synechococcus sp. NIES-970
 Leptolyngbya sp. PCC 7376
100
 Cyanobacterium stanieri PCC 7202
 Cyanobacterium sp. HL-69
100
 Geminocystis sp. NIES-3709
 Geminocystis herdmanii PCC 6308
 Cyanobacterium aponinum PCC 10605
 Prochloron didemni P1-Palau
 Spirulina subsalsa PCC 9445
 Spirulina major PCC 6313
 Filamentous cyanobacterium ESFC-1
 Halothece sp. PCC 7418
 Dactylococcopsis salina PCC 8305
 Cyanobacteria bacterium SW 9 44 58
 Cyanobacteria bacterium QS 4 48 99
 C. bacterium QS 8 64 29
 Rubidibacter lacunae KORDI 51-2
98
B2-a
B2-b
Clade B2
B2-c
30
 Phormidium sp. HE10JO
 Planktothrix serta PCC 8927
 Planktothrix sp. PCC 11201
 Planktothrix prolifica NIVA-CYA 406
 Planktothrix tepida PCC 9214
 Limnospira indica PCC 8005
 Arthrospira sp. TJSD092
 Lyngbya sp. PCC 8106
 Trichodesmium erythraeum IMS101
 Microcoleus vaginatus FGP-2
 Tychonema bourrellyi FEM_GT703
 Kamptonema sp. PCC 6506
 Oscillatoria acuminata PCC 6304
 Geitlerinema sp. PCC 9228
 Geitlerinema sp. PCC 7105
89
100
100
100
100
100
98
100
100
100
44
52
41
100
B2-d
67
100
 Desertifilum sp. IPPAS B-1220
 Roseofilum reptotaenium AO1-A
A
H
100
100
100
100
71
95
100
100
70
95
99
 Leptolyngbya sp. JSC-1
 Elainella saxicola E1
 Leptolyngbya ohadii IS1
 Leptolyngbya sp. O-77
 Leptolyngbya sp. NIES-3755
 Leptolyngbya boryana IAM M-101
 Alkalinema sp. CACIAM 70d
 Phormidesmis priestleyi ULC007
 Oscillatoriales cyanobacterium JSC-12
 Leptolyngbya frigida ULC18
 Leptolyngbya sp. 'hensonii'
 Neosynechococcus sphagnicola sy1
D
 Geitlerinema sp. PCC 7407
38
100
 Limnothrix sp. PR1529
 Oscillatoriales cyanobacterium PH2015 14S 56 116
C1-a
100
45
53
100
65
62
100
67
100
100
100
100
100
99
100
100
100
100
100
100
100
100
100
100
100
100
100
100
100
100
100
 Synechococcus sp. WH 8109
 Synechococcus sp. KORDI-52
 Synechococcus sp. CC9902
 Synechococcus sp. WH 8102
 Synechococcus sp. KORDI-49
 Synechococcus sp. KORDI-100
 Synechococcus sp. WH 8016
 Synechococcus sp. WH 8020
 Synechococcus sp. WH 7803
 Synechococcus sp. RS9916
 Synechococcus sp. RS9917
 Prochlorococcus marinus MIT1312
 Prochlorococcus marinus MIT9303
 Prochlorococcus marinus MIT9301
1/4 of original
length
 Prochlorococcus sp. RS01
 Prochlorococcus marinus AS9601
 Prochlorococcus marinus MED4
 Prochlorococcus marinus NATL1A
 Prochlorococcus sp. MIT0801
 Prochlorococcus marinus CCMP1375
1/3 of ori-
ginal length
 Prochlorococcus sp. MIT0602
 Prochlorococcus marinus MIT9211
 Cyanobium gracile PCC 6307
 Aphanothece cf. minutissima CCALA 015
 Synechococcus sp. WH 5701
 Synechococcus sp. SynAce01
 Cyanobium sp. PCC 7001
 Cyanobium sp. NIES-981
 Synechococcus sp. CB0101
 Vulcanococcus limneticus LL
 Synechococcus sp. RCC307
Synechococcus lacustris Tous
 Candidatus Synechococcus spongiarum 15L
42
63
100
100
96
99
100
 Thermosynechococcus elongatus BP-1
 Synechococcus lividus PCC 6715
 Synechococcus sp. PCC 6312
 Cyanothece sp. PCC 7425
 Acaryochloris marina MBIC11017
 Acaryochloris sp. RCC1774
67
94
 Nodosilinea nodulosa PCC 7104
 Halomicronema hongdechloris C2206
100
100
 Leptolyngbya sp. PCC 7375
100
 Leptolyngbya sp. Heron Island J
100
 Phormidesmis priestleyi Ana
 Synechococcus sp. PCC7335
80
100
1/3 of original
length
 Gloeomargarita lithophora Alchichica-D10
100
100
 Synechococcus elongatus PCC 7942
 Synechococcus sp. UTEX 2973
100
 Prochlorothrix hollandica PCC 9006
0.10
90
100
62
42
100
100
 Pseudanabaena sp. PCC 6802
 Synechococcus sp. PCC 7502
 Pseudanabaena frigida ULC066 bin1
 Pseudanabaena biceps PCC 7429
 Pseudanabaena sp. PCC 7367
100
100
 Synechococcus sp. JA-3-3Ab
 Synechococcus sp. JA-2-3B‘a(2-13)
 Synechococcus sp. PCC 7336
100
 Gloeobacter violaceus PCC 7421
 Gloeobacter kilaueensis JS1
Clade C1
 Prochlorococcus marinus CCMP1986
C1-b
C1-c
C2
C3
E
F
Figure S2. Phylogenetic MLSA analysis of 213 taxa. The tree that is based on 43 universal proteins with 6,458 amino acid positions is representing the cyanobacterial diversity. The maximum likelihood tree was inferred by RAxML under a GTR+4 model, including 100 bootstrap replicates. The tree is divided into eight monophyletic clades (A-H), of which B and C are further divided into the subclades B1-B4 and C1-C3. Distinct lineages of B1, B2 and C1 are moreover highlighted with -a, -b and -c. The three non-axenic strains, whose metagenome was analyzed in this study, are shown in red and highlighted in yellow. Note, only four deeply diverging taxa, probably representing ancient and diverse lineages, are currently not part of one of these clades. The scale bar indicates the mean number of inferred non-synonymous substitutions per amino acid position.
G
