# Supplementary material for: Filling the Gaps in the Cyanobacterial Tree of Life—Metagenome Analysis of Stigonema ocellatum DSM 106950, Chlorogloea purpurea SAG 13.99 and Gomphosphaeria aponina DSM 107014
Source: Genes (Basel). 2021 Mar 9;12(3):389. doi: 10.3390/genes12030389 (PMC8001431; doi:10.3390/genes12030389)
Supplement: Supplementary file 1 [file genes-12-00389-s001.zip › genes-1023673-SI/B_Supplemental-Data_Revision_Marter-et-al-210203/FigureS3_Cyano-B1-70_CheckM_5_20_R238_B1_R70_V3711_RaxGTR4g_210129.pptx]

## Slide 1
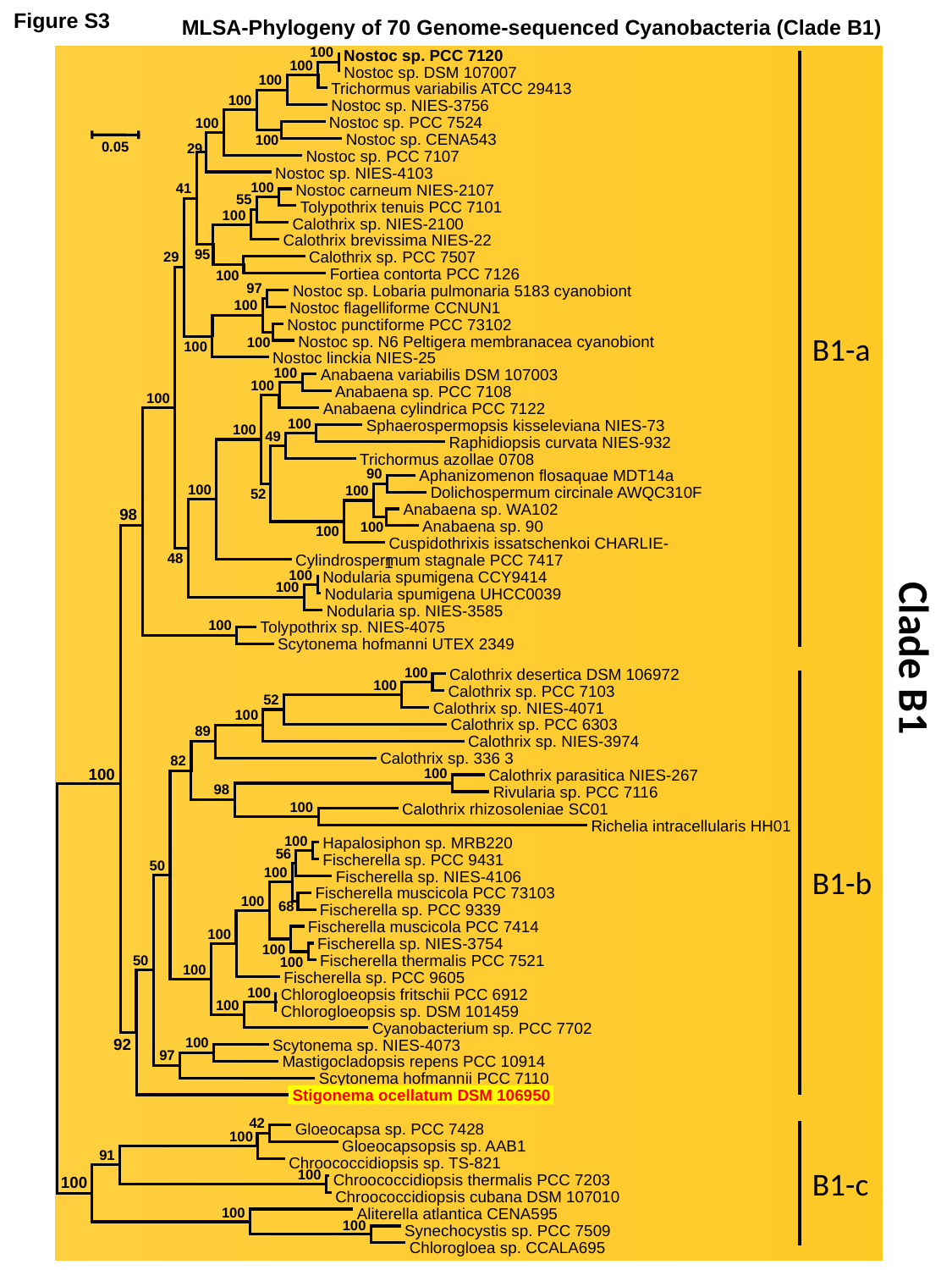

MLSA-Phylogeny of 70 Genome-sequenced Cyanobacteria (Clade B1)
Figure S3
100
100
100
100
100
100
29
100
41
55
100
95
29
100
97
100
100
100
100
100
100
100
100
49
90
100
100
52
98
100
100
48
100
100
100
100
100
52
100
89
82
100
100
98
100
100
56
50
100
100
68
100
100
50
100
100
100
100
100
92
97
42
100
91
100
100
100
100
 Nostoc sp. PCC 7120
 Nostoc sp. DSM 107007
 Trichormus variabilis ATCC 29413
 Nostoc sp. NIES-3756
 Nostoc sp. PCC 7524
 Nostoc sp. CENA543
 Nostoc sp. PCC 7107
 Nostoc sp. NIES-4103
 Nostoc carneum NIES-2107
 Tolypothrix tenuis PCC 7101
 Calothrix sp. NIES-2100
 Calothrix brevissima NIES-22
 Calothrix sp. PCC 7507
 Fortiea contorta PCC 7126
 Nostoc sp. Lobaria pulmonaria 5183 cyanobiont
 Nostoc flagelliforme CCNUN1
 Nostoc punctiforme PCC 73102
 Nostoc sp. N6 Peltigera membranacea cyanobiont
 Nostoc linckia NIES-25
 Anabaena variabilis DSM 107003
 Anabaena sp. PCC 7108
 Anabaena cylindrica PCC 7122
 Sphaerospermopsis kisseleviana NIES-73
 Raphidiopsis curvata NIES-932
 Trichormus azollae 0708
 Aphanizomenon flosaquae MDT14a
 Dolichospermum circinale AWQC310F
 Anabaena sp. WA102
 Anabaena sp. 90
 Cuspidothrixis issatschenkoi CHARLIE-1
 Cylindrospermum stagnale PCC 7417
 Nodularia spumigena CCY9414
 Nodularia spumigena UHCC0039
 Nodularia sp. NIES-3585
 Tolypothrix sp. NIES-4075
 Scytonema hofmanni UTEX 2349
0.05
B1-a
Clade B1
 Calothrix desertica DSM 106972
 Calothrix sp. PCC 7103
 Calothrix sp. NIES-4071
 Calothrix sp. PCC 6303
 Calothrix sp. NIES-3974
 Calothrix sp. 336 3
 Calothrix parasitica NIES-267
 Rivularia sp. PCC 7116
 Calothrix rhizosoleniae SC01
 Richelia intracellularis HH01
 Hapalosiphon sp. MRB220
 Fischerella sp. PCC 9431
 Fischerella sp. NIES-4106
 Fischerella muscicola PCC 73103
 Fischerella sp. PCC 9339
 Fischerella muscicola PCC 7414
 Fischerella sp. NIES-3754
 Fischerella thermalis PCC 7521
 Fischerella sp. PCC 9605
 Chlorogloeopsis fritschii PCC 6912
 Chlorogloeopsis sp. DSM 101459
 Cyanobacterium sp. PCC 7702
 Scytonema sp. NIES-4073
 Mastigocladopsis repens PCC 10914
 Scytonema hofmannii PCC 7110
 Stigonema ocellatum DSM 106950
B1-b
 Gloeocapsa sp. PCC 7428
 Gloeocapsopsis sp. AAB1
 Chroococcidiopsis sp. TS-821
 Chroococcidiopsis thermalis PCC 7203
 Chroococcidiopsis cubana DSM 107010
 Aliterella atlantica CENA595
 Synechocystis sp. PCC 7509
 Chlorogloea sp. CCALA695
B1-c
