# Supplementary material for: Filling the Gaps in the Cyanobacterial Tree of Life—Metagenome Analysis of Stigonema ocellatum DSM 106950, Chlorogloea purpurea SAG 13.99 and Gomphosphaeria aponina DSM 107014
Source: Genes (Basel). 2021 Mar 9;12(3):389. doi: 10.3390/genes12030389 (PMC8001431; doi:10.3390/genes12030389)
Supplement: Supplementary file 1 [file genes-12-00389-s001.zip › genes-1023673-SI/B_Supplemental-Data_Revision_Marter-et-al-210203/Supplemental-Textfiles_S1-S2_Classification-of-metagenomic-bins_210127.pdf]

Supplemental Text Files

S1. Text mining as analysis tool for metagenomes (Illumina)

S1A *Quality control of metagenome assemblies via text mining*

The text mining approach served as a very helpful analytical tool for the quality control of metagenome data that were either established in our own laboratory or retrieved from public databases. We initially detected a typical bioinformatic artifact resulting from a missing filtering step of non-informative Illumina primer-dimer reads. Binning and text mining of the unfiltered Illumina sequences recurrently resulted in the misleading key words '*Cyprinus*', '*carpio*' or 'carp' and the outcome was hence designated as the "carp artifact". These hit subjects were traced back to one of the five available eukaryotic draft genomes of the European carp *Cyprinus carpio* in the NCBI database (assembly: GCA_000951615.2), which is indicative of a massive quality problem in its submission. Accordingly, text mining as an internal control allowed us to overcome the "carp artifact" by removing primer-dimer sequences originating from the Illumina library preparation.

S1B *Identification of separate 'plasmid' bins via text mining*

Our text mining approach enables an initial phylogenetic assessment of those bins that obtained the marker lineage 'root' due to the lack of any marker proteins from the set of reference sequences (0.00% completeness, 0.00% contamination). A prime example is the most abundant bin01 from *C. purpurea* that very likely comprises extrachromosomal replicons (ECRs) of the cyanobacterium as indicated by the keyword 'plasmid' (Table S4). Moreover, the coverage ratio of 1.7 (1079× [bin01] *versus* 622× [bin02]) indicates that the putative cyanobacterial plasmids are low-copy number replicons. With a size of 394 kb this portion of cyanobacterial DNA comprises 8% of the total *Chlorogloea* draft genome (4,749 kb) and its assembly into a separate bin is likely the consequence of the higher coverage and a deviant DNA signature compared to those of the chromosome [1]. The deposited draft

genome of *C. purpurea* SAG 13.99 was thus manually adjusted and finally comprises both bins (see Materials and Methods; JADQBB000000000).

S2. Classification of metagenomic bins from non-axenic cyanobacteria

S2A *BLASTN analyses with 16S-rDNA sequences*

The 16S-rRNA, which already provided new insights into the hidden bacterial biodiversity in our cyanobacterial metagenomes (see paragraph 3.5.), should theoretically also allow to precisely determine the closest related relative of each bin. However, the actual results documented the limitations of this marker for a reliable classification of metagenomic bins assembled from Illumina short read sequences (Table S2). Between 44% and 75% of the bins of the three datasets are even lacking any 16S-rDNA sequence, which can be explained by binning problems due to a deviant nucleotide composition and the presence of multiple copies of the rRNA operon [2]. Furthermore, the comparison of identified 16S-rRNA sequences with the respective marker lineage revealed several binning errors. One example is the verrucomicrobial bin21 from *C. purpurea* that contains in addition to the authentic 16S-rRNA gene further alphaproteobacterial sequences from four different families (Table S2). The 16S-rDNA sequence of *S. ocellatum* is correctly located in the cyanobacterial bin01, but this dataset also contains two additional 16S sequences from *Alphaproteobacteria* and *Cytophagia* representing wrongly binned contaminations. The cyanobacterial bins of *C. purpurea* (bin02) and *G. aponina* (bin03) are lacking a 16S-rRNA gene and the respective sequences were wrongly located in bin34 and bin02, respectively. Due to the high quality of the cyanobacterial bin of *C. purpurea* (99.56% completeness, 0.29% contamination) this observation seems paradoxical at first glance, but the quality estimation of CheckM is exclusively based on the presence of a set of universal marker proteins. Accordingly, our analyses of three different low complexity communities clearly show that the 16S-rDNA is no

suitable marker for a reliable taxonomic assessment of metagenome bins derived from high-throughput Illumina sequencing.

S2B *BLASTP analyses with RpoB sequences*

We chose the derived protein sequences of the beta subunit of the DNA-dependent RNA polymerase (RpoB) as a proxy to identify the closest related genomes of our 116 bins (Table S3). The RpoB, which is essential for bacteria, archaea and eukaryotes, represents with a size of at least 1,100 amino acid positions a well-suited marker for rapid phylogenetic analyses [3,4], and it has been exemplified for cyanobacteria that the RpoB allows in comparison with the 16S-rRNA gene a significantly better taxonomic resolution [5]. In the current study metagenome analyses of three non-axenic cyanobacteria showed the usefulness of this marker for the assessment of microbial communities. Only a comparably small number of 9% to 27% of the bins is lacking the RpoB encoding genes and the occurrence of partial sequences is usually restricted to bins with low abundance i.e. with a genome coverage below 5× (Table S3). Two partial RpoB sequences from the *S. ocellatum* bin10 obtained the same best BLASTP hit, i.e. the rhizobium *Labrys okinawensis* RP1T (WP_105865686.1), and both sequences can be manually merged into a complete protein sequence of 1,373 amino acid positions based on an overlap of 47 identical positions (highlighted in ocher, Table S3). A comparable complementation was also possible for the rhizobial RpoB from *Devosia* sp. in *S. ocellatum* bin04 (1,382aa), but the C-terminus was surprisingly found in bin05, which reflects the considerable contamination level of 33.89% in this bin. A plausible explanation for the observed mis-binning is a comparable, i.e. 220- to 245-fold, genome coverage of four bins (bin03 to bin06) representing three abundant *Alphaproteobacteria* and one *Cytophagia*. However, all but one of the complementing partial RpoB proteins (5/6) were located on different contigs of the same bin, which documents the reliability of the metagenomic binning approach. Accordingly, and in clear contrast to the 16S-rDNA (Table S2), the BLASTP

results of complete RpoB sequences are largely consistent with the taxonomic affiliation of the predicted maker lineage (Table S3). The RpoB is hence an excellent marker to identify the closest genome-sequenced reference strain(s). One example is the *S. ocellatum* RpoB of bin03 that shows 99.30% amino acid identity with *Spirosoma fluviale* (*Cytophagaceae*) and the *G. aponina* RpoB of bin05 is even identical to those of *Brevundimonas fluminis* (*Caulobacteraceae*). However, the interpretation of RpoB protein identities for a taxonomic classification is difficult, because general thresholds for the delineation of species, genera, families and higher taxonomic ranks as established for the 16S-rDNA [6,7] are not available for this protein marker. An alternative *rpoB*-based delineation of species and genera on nucleotide level [8,9] might be biased by deviant G+C contents.

Two bins in our datasets, which retrieved the same best BLAST hit by both markers, are of special interest, because they allowed us to calibrate the respective identity values. First, the BLAST results for the alphaproteobacterial bin04 from *G. aponina* revealed *Hyphomonas* sp. CACIAM 19H1 as best hit (RpoB: 92.39%; WP_114104833.1; 16S-rDNA: 97.94%, CP016437.1), thereby proposing that the respective bacteria represent different species of the genus *Hyphomonas*. A second example is bin16 from *C. purpurea* that independently revealed *Luteitalea pratensis* DSM 100886, a member of the subdivision 6 *Acidobacteria* [10,11], as the best hit. The RpoB amino acid identity of 95.51% (WP_110173172.1) corresponds to a 16S-rDNA nucleotide identity of 99.22% (CP015136.1), which clearly documents that the cyanosphere of *Chlorogloea* comprises another strain of the species *L. pratensis* [7]. Accordingly, a comparably conservative RpoB amino acid identity threshold of 96% should allow to rapidly allocate different (meta-)genomes to the same bacterial species. With respect to the three metagenomes investigated in the current study at least 37 of 110 non-cyanobacterial bins are thus representing the same species like their closest genome-sequenced relative (Table S3).

S2C *Text mining with all contigs via BLASTN*

We developed a new BLASTN-based text mining approach to rapidly assess the taxonomic affiliation of the metagenomic bins of non-axenic cyanobacteria. This method identifies the closest relatives of each contig via BLASTN searches in the NCBI nt database and calculates the 'word frequencies' of the top hits for each bin (see Materials and Methods). Accordingly, this complementary method provides additional compositional insights especially for incomplete bins where the phylogenetic marker is missing and for bins with a considerable contamination level. The text mining provides for each bin a list with the 14 most abundant keywords including their numerical quantity (Table S4), which comprises an overview about closely related genomes on genus, species and strain level as well as structural information regarding the genomic localization ('chromosome', 'plasmid'). In bins with a low contamination level the occurrence of different keywords regarding the species or even the genus usually reflects a considerable evolutionary distance to the closest genome-sequenced relative(s). Accordingly, the authentic cyanobacterial bin01 of *S. ocellatum* representing the first genome-sequenced strain of the family *Stigonemataceae* retrieved the genera *Nostoc*, *Calothrix* and *Scytonema* as best hits (Table S4), which is in agreement with their common phylogenetic branching in the order *Nostocales* (see below). The alphaproteobacterial bin02 of the same metagenome revealed *Sphingomonas* as first hit on genus level (477 counts) followed by *Sphingopyxis* and *Sphingobium* with 107 and 46 counts, respectively. The finding of different genera within the *Sphingomonadaceae* confirms the comparably low RpoB sequence identity of 88.75% with *Sphingomonas changbaiensis* as closest genome-sequenced relative (Table S3), thereby showing that a reliable taxonomic classification of this bin is restricted to the family level. In contrast, our text mining approach revealed the genus *Spirosoma* for bin03 and *Devosia* for bin04, which is in agreement with the outcome of the RpoB results exhibiting sequence identities of more than 99.30% with the best hit. Accordingly, the holistic analysis of the metagenomic bins provides reliable information

about the closest related genome(s) of each bin and their most abundant contaminations. However, the diagnostic depth of the BLASTN-based text mining, which was developed for a rapid taxonomic assessment of metagenomes, is limited to the genus level.

References

1. Harrison, P.W.; Lower, R.P.J.; Kim, N.K.D.; Young, J.P.W. Introducing the bacterial "chromid": Not a chromosome, not a plasmid. *Trends Microbiol.* **2010**, *18*, 141–148, doi:10.1016/j.tim.2009.12.010.
2. Roller, B.R.K.; Stoddard, S.F.; Schmidt, T.M. Exploiting rRNA operon copy number to investigate bacterial reproductive strategies. *Nat. Microbiol.* **2016**, *1*, 16160, doi:10.1038/nmicrobiol.2016.160.
3. Case, R.J.; Boucher, Y.; Dahllöf, I.; Holmström, C.; Doolittle, W.F.; Kjelleberg, S. Use of 16S rRNA and rpoB genes as molecular markers for microbial ecology studies. *Appl. Environ. Microbiol.* **2007**, *73*, 278–288, doi:10.1128/AEM.01177-06.
4. Petersen, J.; Wagner-Döbler, I. Plasmid transfer in the ocean - A case study from the roseobacter group. *Front. Microbiol.* **2017**, *8*, 1350, doi:10.3389/fmicb.2017.01350.
5. Will, S.E.; Henke, P.; Boedeker, C.; Huang, S.; Brinkmann, H.; Rohde, M.; Jarek, M.; Friedl, T.; Seufert, S.; Schumacher, M.; et al. Day and night: Metabolic profiles and evolutionary relationships of six axenic non-marine cyanobacteria. *Genome Biol. Evol.* **2019**, *11*, 270–294, doi:10.1093/gbe/evy275.
6. Kim, M.; Oh, H.S.; Park, S.C.; Chun, J. Towards a taxonomic coherence between average nucleotide identity and 16S rRNA gene sequence similarity for species demarcation of prokaryotes. *Int. J. Syst. Evol. Microbiol.* **2014**, *64*, 346–351, doi:10.1099/ijs.0.059774-0.
7. Yarza, P.; Yilmaz, P.; Pruesse, E.; Glöckner, F.O.; Ludwig, W.; Schleifer, K.-H.; Whitman, W.B.; Euzéby, J.; Amann, R.; Rosselló-Móra, R. Uniting the classification

of cultured and uncultured bacteria and archaea using 16S rRNA gene sequences. *Nat. Rev. Microbiol.* **2014**, *12*, 635–645, doi:10.1038/nrmicro3330.

8. Khamis, A.; Colson, P.; Raoult, D.; La Scola, B. Usefulness of rpoB gene sequencing for identification of Afipia and Bosea species, including a strategy for choosing discriminative partial dequences. *Appl. Environ. Microbiol.* **2003**, *69*, 6740–6749, doi:10.1128/AEM.69.11.6740-6749.2003.
9. Adékambi, T.; Shinnick, T.M.; Raoult, D.; Drancourt, M. Complete rpoB gene sequencing as a suitable supplement to DNA-DNA hybridization for bacterial species and genus delineation. *Int. J. Syst. Evol. Microbiol.* **2008**, *58*, 1807–1814, doi:10.1099/ijs.0.65440-0.
10. Huang, S.; Vieira, S.; Bunk, B.; Riedel, T.; Spröer, C.; Overmann, J. First complete genome sequence of a subdivision 6 Acidobacterium strain. *Genome Announc.* **2016**, *4*, 2006–2007, doi:10.1128/genomeA.00469-16.
11. Vieira, S.; Luckner, M.; Wanner, G.; Overmann, J. Luteitalea pratensis gen. nov., sp. nov. a new member of subdivision 6 Acidobacteria isolated from temperate grassland soil. *Int. J. Syst. Evol. Microbiol.* **2017**, *67*, 1408–1414, doi:10.1099/ijsem.0.001827.
